# Genomic virulence markers are associated with severe outcomes in patients with *Pseudomonas aeruginosa* bloodstream infection

**DOI:** 10.1038/s43856-024-00696-4

**Published:** 2024-12-11

**Authors:** John Karlsson Valik, Christian G. Giske, Badrul Hasan, Mónica Gozalo-Margüello, Luis Martínez-Martínez, Manica Mueller Premru, Žiga Martinčič, Bojana Beović, Sofia Maraki, Maria Zacharioudaki, Diamantis Kofteridis, Kate McCarthy, David Paterson, Marina de Cueto, Isabel Morales, Leonard Leibovici, Tanya Babich, Fredrik Granath, Jesús Rodríguez-Baño, Antonio Oliver, Dafna Yahav, Pontus Nauclér

**Affiliations:** 1 Department of Medicine, Solna, Division of Infectious Diseases, Karolinska Institutet, Stockholm, Sweden; 2 Department of Infectious Diseases, Karolinska University Hospital, Stockholm, Sweden; 3 Department of Laboratory Medicine, Karolinska Institutet, Stockholm, Sweden; 4 Department of Clinical microbiology, Karolinska University Hospital, Stockholm, Sweden; 5 Service of Microbiology. Hospital Universitario Marqués de Valdecilla. Instituto de Investigación Marqués de Valdecilla (IDIVAL), Cantabria, Spain; 6 CIBER de Enfermedades Infecciosas-CIBERINFEC (CB21/13/00068), Instituto de Salud Carlos III, Madrid, Spain; 7 Unit of Microbiology, University Hospital Reina Sofía, Córdoba, Spain; 8 Department of Agricultural Chemistry, Soil Science and Microbiology, University of Cordoba, Córdoba, Spain; 9 Maimonides Biomedical Research Institute of Cordoba (IMIBIC), Córdoba, Spain; 10 CIBER de Enfermedades Infecciosas (CIBERINFEC), Instituto de Salud Carlos III, Madrid, Spain; 11 Faculty of Medicine, University of Ljubljana, Ljubljana, Slovenia; 12 Department of Infectious Diseases, University Medical Centre Ljubljana, Ljubljana, Slovenia; 13 Department of Internal Medicine, University Hospital of Heraklion, Crete, Greece; 14 Pathology Queensland, Royal Brisbane and Woman’s Hospital, Brisbane, QLD Australia; 15 University of Queensland Centre for Clinical Research, Brisbane, QLD Australia; 16 Unidad Clínica de Enfermedades Infecciosas y Microbiología, Instituto de Biomedicina de Sevilla (IBiS)/CSIC, Hospital Universitario Virgen Macarena / Departamentos de Medicina y Microbiología, Universidad de Sevilla, Sevilla, Spain; 17 CIBERINFEC, Instituto de Salud Carlos III, Madrid, Spain; 18 Servicio de Urgencias, Hospital Universitario Virgen Macarena, Sevilla, Spain; 19 Research Authority, Rabin Medical Center, Beilinson hospital, Petah-Tiqva, Israel; 20 Department of Medicine, Solna, Division of Clinical Epidemiology, Karolinska Institutet, Stockholm, Sweden; 21 Servicio de Microbiología, Hospital Universitario Son Espases, Instituto de Investigación Sanitaria Illes Balears (IdISBa), CIBERINFEC, Palma de Mallorca, Spain; 22 Infectious Diseases Unit, Sheba Medical Center, Ramat-Gan, Israel

**Keywords:** Clinical microbiology, Prognostic markers, Bacterial pathogenesis, Infectious-disease epidemiology

## Abstract

**Background:**

*Pseudomonas aeruginosa* (PA) bloodstream infection (BSI) is a common healthcare-associated complication linked to antimicrobial resistance and high mortality. Ongoing clinical trials are exploring novel anti-virulence agents, yet studies on how bacterial virulence affects PA infection outcomes is conflicting and data from real-world clinical populations is limited.

**Methods:**

We studied a multicentre cohort of 773 adult patients with PA BSI consecutively collected during 7-years from sites in Europe and Australia. Comprehensive clinical data and whole-genome sequencing of all bacterial strains were obtained.

**Results:**

Based on the virulence genotype, we identify several virulence clusters, each showing varying proportions of multidrug-resistant phenotypes. Genes tied to biofilm synthesis and epidemic clones ST175 and ST235 are associated with mortality, while the type III secretion system is associated with septic shock. Adding genomic biomarkers to machine learning models based on clinical data indicates improved prediction of severe outcomes in PA BSI patients.

**Conclusions:**

These findings suggest that virulence markers provide prognostic information with potential applications in guiding adjuvant sepsis treatments.

## Plain language summary

*Pseudomonas aeruginosa* bacteria are often found in the hospital environment, primarily infecting vulnerable patients with underlying health conditions. Due to antibiotic resistance, which occurs when bacteria are not killed by antibiotic treatment, these infections are often difficult to treat, and death rates are high. In this study, we analyzed data from patients in Europe and Australia with bloodstream infections to understand how bacterial traits affect patient outcomes. Using genetic information from the bacteria, we identified characteristics associated with antibiotic resistance. In addition, we found certain bacterial traits, such as the ability to synthesize toxins and biofilms, were linked to disease severity and mortality risk. These findings indicate that specific characteristics of *P. aeruginosa* may influence the severity of infection and could be targeted in newly developed treatments.

## Abstract (web summary)

Valik et al. incorporate genomic biomarkers from *Pseudomonas aeruginosa* isolates and clinical data into machine-learning models to predict the severity of bloodstream infection. Bacterial virulence markers identified through whole genome sequencing offer prognostic insights that could inform treatment strategies in sepsis management.

## Introduction

Each year sepsis affects 50 million people and is associated with approximately 11 million deaths worldwide, which means that 1 in 5 deaths are due to sepsis^1^. There is an ongoing debate if bacterial virulence, in comparison to host factors, has a major influence on the outcome of sepsis. The emerging crisis of antimicrobial resistance has deleterious consequences for the treatment of sepsis, especially in Gram-negative infections such as *Pseudomonas aeruginosa* (PA). This highly adaptive opportunistic pathogen, which has been listed as a priority bacteria by the World Health Organization, is commonly isolated in hospital-acquired bloodstream infections (BSI) and primarily affects immunocompromised hosts in oncology, pulmonary, or intensive care^2–4^. Mortality is high ranging between 20 and 40% and is affected by both host and treatment factors^5,6^.

The current view is that PA demonstrates a panmictic population structure, characterized by random genetic exchange, disrupted by emerging epidemic clones^7,8^. There is a rising global occurrence of healthcare-associated infections attributable to multidrug-resistant (MDR) or extensively drug-resistant (XDR) PA which is often traced back to a few high-risk clones such as sequence type (ST)111, ST175, and ST235^9–11^. These MDR/XDR clonal complexes are typically defined by having 6 out of 7 identical alleles in multilocus sequence typing (MLST) and often share similar biological attributes, such as increased biofilm formation and mutation frequency, but also reduced motility and fitness^12,13^.

Identification and molecular characterization of clones have previously been difficult and time-consuming, but high-throughput whole-genome sequencing (WGS) methods are now available for routine clinical microbiological laboratories within hours of pathogen detection^14,15^. The PA chromosome consists of a large core genome, which is highly conserved between strains, and an accessory gene pool with elements that can be horizontally transferred and vary among strains^16^. Various components of the accessory genome, including but not limited to PA genomic islands (PAGI) or PA pathogenicity islands (PAPI), contribute to unique phenotypic plasticity and the ability to adapt to diverse environments. These elements are widely dispersed throughout the genome, sometimes forming mobile islands like PAGI/PAPI, and sometimes existing as non-mobile segments. PAGI/PAPI may harbor both virulence factors, such as secretion of exotoxins via type III secretion system (T3SS), and resistance mechanisms, which are suggested to influence outcomes in clinical infections^16–18^. It is probable that certain virulence features necessitate complex interactions among multiple genomic islands, and it has also been demonstrated that more virulent PA strains carry PAGIs that are not present in less virulent counterparts^19^. Treatments targeting virulence factors, such as biofilm formation or the T3SS, hold promise as adjuvant therapies for PA infections. However, most of these agents are still in the early stages of clinical development, and their potential role in PA BSI remains unclear^20–22^.

As of today, few studies have investigated the impact of virulence factors in clinical invasive PA isolates^23^. Peña et al. examined a Spanish cohort of clinical cases of PA BSI and found that the *exoU* genotype of the T3SS was associated with increased 5-day mortality^24^. This study was limited to one geographic region, warranting further research to validate their findings, but also to explore if more extensive virulence genotyping reveals other bacterial factors related to disease severity in clinical PA infection. The aim of this study was to evaluate the relationship between bacterial genomic virulence markers and patient characteristics, septic shock at blood culture collection, and mortality in PA BSI. Our cohort includes 773 adult patients with PA BSI, consecutively collected from multiple sites in Europe and Australia. Based on the bacterial genotype, we identify several virulence clusters, each showing varying proportions of multidrug resistance. Genes linked to biofilm synthesis and epidemic clones ST175 and ST235 correlate to mortality, while the T3SS is associated with septic shock. Overall, our findings indicate that genomic markers offer prognostic information in severe PA infections, highlighting how WGS data can improve diagnostic accuracy in sepsis.

## Methods

### Design, setting, and study population

The study was approved by the Regional Ethical Review Board in Stockholm (approval number 2015/1184-31 and 2022-02595-02) and performed in accordance with the permission. According to national standards for similar studies, the Regional Ethical Review Board in Stockholm gave their approval to the study with a waiver of consent from participants. Each participating center was required to have local ethical review board approval or comply with other local regulatory requirements for collecting data for research purposes. The study had a retrospective cohort design and included consecutive adult (≥18 years) patients with PA BSI and available PA clinical isolates from 6 sites in Europe and Australia (Seville, Santander, Heraklion, Ljubljana, Stockholm, and Brisbane) between year 2009-2015 (Figure [Fig]. 1). Centers were recruited from an international PA BSI research network^6,25,26^. The centers included in this study contributed data during time periods when they routinely saved all PA BSI strains (Supplementary Table 1). Polymicrobial bloodstream infections were excluded (in case other bacteria and fungi were regarded clinically significant, as decided by investigators). A single co-isolation of coagulase-negative staphylococci, *Micrococcus spp*, *Bacillus spp*, or viridans streptococci was not considered as a reason for exclusion. If recurrent PA BSI episodes were present, only the first episode per patient was included.

**Fig. 1:**
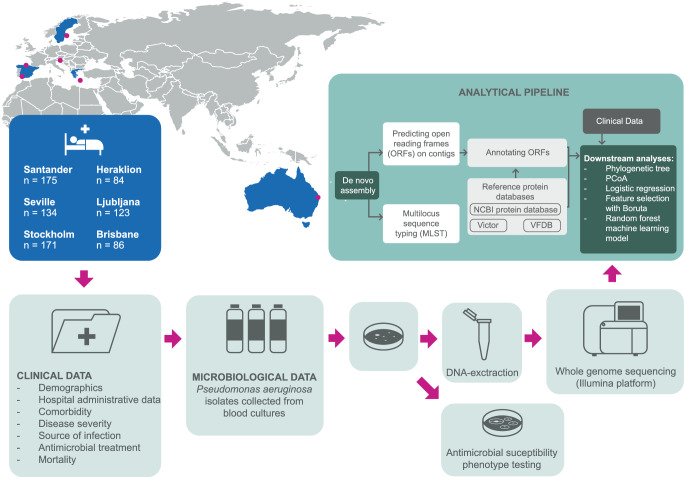
Schematic overview of the study. The figure illustrates the study design, setting, study population, and analytical pipeline. It was created by the authors using Adobe Illustrator and R. Open Reading Frames (ORFs), Multilocus Sequence Typing (MLST), The National Center for Biotechnology Information (NCBI), Virulence Factor Database (VFDB), and Principal Coordinate Analysis (PCoA).

### Clinical data collection and definitions

Patient-specific variables were collected by review of medical records at each participating center according to a common electronic case report form (eCRF). The assessors of clinical information were blinded to the results of WGS. Variables included demographics, hospital administrative data, prior surgery, clinical management, and the Charlson comorbidity index (CCI)^27,28^. Immunocompromised state was defined as either chemotherapy during the last 30 days, systemic corticosteroid treatment (>10 milligrams of prednisone for >29 days), neutropenia (absolute neutrophil count <0.5 × 10^9^/liter), solid organ transplant, bone marrow transplant and/or chronic dialysis treatment. Place of acquisition was classified as community, healthcare-associated, or nosocomial according to Friedman et al. ^29^. Source of infection was determined guided by the Center for Disease Control and Prevention (CDC) criteria^30^. Vascular catheter-associated infection was defined either as BSI and peripheral line phlebitis with no other source, BSI and presence of a central line with no other source, or according to the 2009 guidelines of the Infectious Diseases Society of America^31^. Appropriate empiric therapy was defined as receiving antimicrobial therapy to which the PA isolate was in vitro susceptible before the culture result was known. Septic shock was defined as sustained hypotension despite adequate fluid replacement and the need for starting or increasing the dosing of vasopressor drugs. Information on septic shock was unavailable from Seville (*n* = 134). Length of hospital stay and date of death were collected for all patients.

### Bacterial isolates collection and antimicrobial susceptibility testing

Bacterial isolates were identified from the clinical microbiological laboratories at each study site and sent to Karolinska University Hospital, Stockholm, Sweden for further characterization. Antimicrobial susceptibility testing (AST) for the antimicrobial agents ceftazidime, aztreonam, piperacillin/tazobactam, meropenem, imipenem, tobramycin, amikacin, gentamycin, and ciprofloxacin was performed according to the European Committee on Antimicrobial Susceptibility Testing (EUCAST) disk diffusion method using Mueller-Hinton agar (Thermo Fischer Scientific, MA, USA) plates and disks from Oxoid (Basingstoke, UK). The disk diffusion method has been extensively calibrated to the reference broth microdilution (https://www.eucast.org/ast_of_bacteria/calibration_and_validation). Interpretation of susceptibility was based on EUCAST clinical breakpoint tables version 10.0 and control strains *Pseudomonas aeruginosa* ATCC 27853 and *Escherichia coli* ATCC 25922 were employed to verify the quality of the AST method. Multidrug resistance (MDR) was defined as phenotypic resistance to three or more antimicrobial drugs from different drug classes and extensively drug-resistant (XDR) was defined as phenotypic resistance to at least one agent in all antimicrobial categories^32^. The material resources are listed in Supplementary Table 2.

### Whole-genome sequencing

Bacterial colonies were collected from overnight cultures for DNA extraction with the EZ1 Advanced XL system (Qiagen, Hilden, Germany) according to the manufacturer's instructions. The quantity of the extracted DNA was measured using a Qubit™ 3.0 fluorometer with the double-stranded DNA (dsDNA) assay kit (Thermo Fischer Scientific, MA, USA). Extracted DNA was diluted to an approximate target sample concentration of 10 nanogram/microliters and a target sample volume of 50 microliters before being sequenced on Illumina HiSeq 2500 system (San Diego, CA, USA) at Science for Life Laboratory (SciLifeLab, Solna, Sweden), producing 2 × 150 short paired-end sequences. The investigators performing all laboratory analyses were blinded to patient characteristics and outcomes.

### Bioinformatics analyses

Sequencing quality control was assessed through the in-house bioinformatic pipeline microSALT (https://github.com/Clinical-Genomics/microSALT) and isolates deemed of insufficient quality were re-sequenced (Supplementary Table 3). In addition, the quality of the reads was subsequently assessed using the FastQC program^33^. Paired-end reads with low-quality bases were trimmed to reach a score of 20, and single reads with less than 20 bases in length were filtered using Trim Galore^34^. The remaining paired-end and single reads were assembled de novo into longer contigs using SPAdes with the *careful* setting^35,36^. No filtering of contigs was performed. The sequence typing (ST) of the isolates was identified as follows: First, the data related to *P. aeruginosa* were downloaded from the databases of allelic profiles and sequences on pubMLST^37^. Then, using the BLASTn algorithm in the BLAST+ package, contigs were searched against the genes: *acsA*, *aroE*, *guaA*, *mutL*, *nuoD*, *ppsA*, *trpE*^38^. The perfect match and complete set of genes were assessed for each isolate, and the corresponding ST was identified. The geographical distribution of STs was visualized with the circlize package^39^. To identify the phylogenetic tree of the discovered STs, all recovered genes in the allelic profile were aligned separately using multiple sequencing alignment tools MAFFT, and the seven alignment results were concatenated^40^. Then the phylogenetic tree was calculated using the maximum-likelihood algorithm in FastTree and visualized using the ggtree package in R^41–43^. Open reading frames (ORFs) on contigs were predicted using Prodigal, and Diamond BLASTx was used to search ORFs against reference proteins in the Virulence Factor Database (VFDB), Victor database, and The Comprehensive Antibiotic Resistance Database (CARD)^44–48^. In the main analyses, the coverage and identity thresholds were set at 80% to be considered a match. Sensitivity analyses with 95% identity and 80% coverage thresholds were performed to evaluate the impact of genetic variation. Since VFDB and Victor databases contain overlapping features, annotations were filtered to exclude redundancies. The bioinformatic software is listed in Supplementary Table 2.

### Data processing and virulence clusters

After annotation of virulence genes, there were 247 sequences matching reference proteins in the VFDB and 91 sequences matching reference proteins in the Victors database. To explore clusters of virulence genotypes in the cohort, we performed Principal Coordinate Analysis (PCoA) based on a Euclidean distance matrix of the binary detected/not detected of all annotated virulence genes from the VFDB. Non-overlapping virulence clusters were identified using the unsupervised k-means clustering algorithm, applied to positions obtained from the PCoA. This approach allows for a reduced-dimensional representation of the data while capturing the major variations among samples. The optimal number of clusters (k) was determined using the elbow method, which identifies the point at which adding additional clusters results in a lesser reduction in the sum of the squared distances from each data point to the centroid of its cluster (within-cluster sum of squares [WCSS]) (Supplementary Fig. 1).

**Fig. 2:**
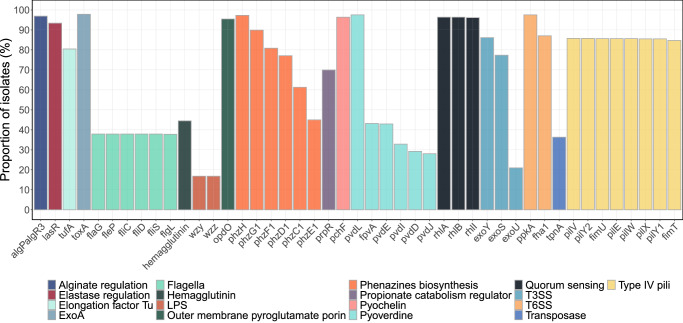
Distribution of virulence genes and function. Bar chart showing the level of collinearity between virulence genes based on function and occurrence in the 773 isolates. A gene identity threshold of 80% was used. Lipopolysaccharides (LPS), type III secretion system (T3SS), and type VI secretion system (T6SS).

Since a large portion of virulence genes were either detected or not detected in most of the bacterial isolates, they were filtered based on frequency to enable meaningful downstream analyses of individual genes. A threshold of virulence gene detection in more than 2%, but less than 98% of the isolates was chosen. This resulted in 40 of 247 genes from the VFDB, 5 of 91 genes from the Victors database, and 33 of 123 genes from the CARD database after excluding duplicate genes. After reviewing gene annotation, an additional 2 genes were removed from further analyses due to known difficulties with misclassification in gene annotation (*wzy* and *wzz*)^49^. To improve model stability and interpretability, virulence genes coding for the same virulence factor which also showed widespread collinearity (flagella [*flaG, fleP, flgL, fliC, fliD*, *fliS*], quorum sensing [*rhlA, rhlB, rhlI*] and type IV pili [*fimT, fimU, pilE, pilV, pilW, pilX, pilY1, pilY2*]) were further grouped resulting in totally 26 gene variables (Fig. 2).

### Statistics and Reproducibility

Differences in age distribution and disease proportions were evaluated using the Kruskal-Wallis test, Chi-square test, or Fisher’s exact test for multiple groups with simulated *p*-values based on 10,000 replicates, as appropriate. If the global test was statistically significant, contrasts between the most extreme values were compared with all the other epidemic clones using the Wilcoxon rank sum test, Chi-square test, or Fisher’s exact test, as appropriate. In the second step, we evaluated the association of epidemic clones, virulence clusters, and individual virulence genes with patient outcomes. Each virulence trait was first assessed in an univariable logistic regression model. Furthermore, ST and virulence clusters, T3SS, as well as individual virulence genes with a *p*-value < 0.1 in the univariate model, were then assessed in a multivariable logistic regression model. To improve statistical power, the largest cluster was used as a reference. Possible confounders were identified based on prior knowledge using a directed acyclic graph and the models were finally adjusted for geographical site, age group, sex, Charlson comorbidity index group, immunosuppressed state, department of hospitalization, and nosocomial infection (Supplementary Fig. 2). The final cohort consisted of *n* = 772 episodes due to missing data on the department of hospitalization for 1 patient. When assessing the septic shock outcome, Spain (Seville) was excluded due to missing data, and the model was based on *n* = 638 episodes. Confidence intervals (CI) are presented at the 95% level. Two-sided *P*-values ≤ 0.05 were considered statistically significant. All analyses were performed in R version 4.3.1 using the suite of packages from the Tidyverse.

### Machine learning model

The cohort was randomly split 80/20 into a training set for model development and tuning, and a test set for model assessment. Feature selection was performed using a Boruta algorithm in the training set^50^. The algorithm is designed to create a random shadow feature for each independent variable. It then fits a random forest classifier and iteratively removes independent variables that are less important than the random features based on statistical testing. After this, several random forest classifiers were trained to predict each outcome using the Caret package and Ranger function in R. Each model was based on a different set of variables: (Reference model) patient characteristics alone (age, sex, comorbidity, department of hospitalization, immunosuppressed state and nosocomial infection), (Model 2) combination of patient characteristics and all filtered and grouped virulence genes (*n* = 29), (Model 3) combination of patient characteristics and epidemic clones occurring >5 times (*n* = 25), (Model 4) combination of patient characteristics and epidemic clones occurring >10 times (n = 11), (Model 5) combination of patient characteristics and virulence clusters A–K, (Model 6) combination of patient characteristics and virulence genes selected by the Boruta algorithm, and (Model 7) combination of patient characteristics with virulence genes and resistance genes selected by the Boruta algorithm. To avoid overfitting, 8-fold stratified cross-validation was used during training. Model training was further improved by using the Synthetic Minority Oversampling Technique (SMOTE) to handle outcome class imbalance. The number of trees was set to 1000 and hyperparameter tuning was performed for mtry and splitrule. Minimum node size was held constant at 1 throughout the tuning. To account for the randomness of the 80/20 data split and provide a more reliable measure of model performance, we repeated the process 1000 times using different random seeds. Prediction model outputs were shown as a distribution of the area under the receiver operating characteristics curves (AUROC) for the 1000 different splits in the validation set. The difference in AUROC was determined by subtracting the AUROC of the Reference model from each of the models that included virulence data (Models 2–6). This calculation was performed across all 1000 data splits. Since the analyses were exploratory, no specific definition of test positivity was applied. All analyses were performed with R version 4.3.1.

### Reporting summary

Further information on research design is available in the Nature Portfolio Reporting Summary linked to this article.

## Results

### Characteristics of the study population

During the study period, 836 patients suffered PA BSI and 773 bacterial isolates were available for WGS (Fig. 1 and Supplementary Table 1). Characteristics of all patients at the time of inclusion in the study are presented in Table 1. In total, 267 of 773 (34.5%) patients were females, the median age was 68 years (interquartile range [IQR] 57–78 years), and 571 of 773 (73.8%) patients had a Charlson Comorbidity Index (CCI) index of 2 points or more, indicating a high burden of comorbidities. Of the 773 patients in the cohort, 131 (16.9%) had no underlying comorbidities registered, 138 (17.9%) had chronic lung disease, and 577 (47.1%) were immunocompromised. Most patients suffered a nosocomial BSI (410 of 773, 53.0%), and the department of hospitalization at BSI onset was often medical wards (62.0%), followed by Intensive Care Units (ICU) (22.5%) and surgical wards (15.4%). Counting from blood culture collection, the 7-day mortality rate was 15.5% (120 of 773) and the 30-day mortality rate was 23.5% (182 of 773). Septic shock was registered at the onset of bloodstream infection (BSI) in 115 out of 639 patients (18.0%; data from Seville excluded due to incompleteness). The 7-day mortality rate for this group was 44.4% (51 out of 115 patients), and the 30-day mortality rate was 53.9% (62 out of 115 patients).

**Table 1:** Clinical characteristics of the study participants

Variable	Overall	Australia	Greece	Spain (Santander)	Spain (Seville)	Slovenia	Sweden
Number of patients	773	86	84	175	134	123	171
Female, *n* (%)	267 (34.5)	32 (37.2)	25 (29.8)	61 (34.9)	42 (31.3)	42 (34.1)	65 (38.0)
Age, Median [IQR]	68.0 [57.0, 78.0]	66.0 [56.0, 79.8]	66.5 [55.8, 75.2]	65.0 [53.0, 78.0]	67.0 [56.0, 77.8]	69.0 [59.5, 79.0]	70.0 [59.0, 77.0]
Age group, *n* (%)							
18–49	103 (13.3)	13 (15.1)	13 (15.5)	30 (17.1)	14 (10.4)	15 (12.2)	18 (10.5)
50–59	134 (17.3)	14 (16.3)	12 (14.3)	37 (21.1)	30 (22.4)	16 (13.0)	25 (14.6)
60–69	180 (23.3)	22 (25.6)	20 (23.8)	37 (21.1)	32 (23.9)	31 (25.2)	38 (22.2)
70–79	196 (25.4)	15 (17.4)	25 (29.8)	33 (18.9)	33 (24.6)	31 (25.2)	59 (34.5)
≥80	160 (20.7)	22 (25.6)	14 (16.7)	38 (21.7)	25 (18.7)	30 (24.4)	31 (18.1)
Charlson, Median [IQR]	2.0 [1.0, 4.0]	2.0 [1.0, 3.0]	2.0 [1.0, 6.0]	2.0 [2.0, 5.0]	2.0 [0.0, 4.0]	2.0 [0.5, 4.0]	2.0 [2.0, 5.0]
Charlson category, n (%)							
0–1	202 (26.1)	35 (40.7)	25 (29.8)	30 (17.1)	47 (35.1)	39 (31.7)	26 (15.2)
2–3	325 (42.0)	38 (44.2)	26 (31.0)	78 (44.6)	52 (38.8)	44 (35.8)	87 (50.9)
4–5	107 (13.8)	10 (11.6)	10 (11.9)	30 (17.1)	16 (11.9)	21 (17.1)	20 (11.7)
≥6	139 (18.0)	3 (3.5)	23 (27.4)	37 (21.1)	19 (14.2)	19 (15.4)	38 (22.2)
Malignant disease, *n* (%)	363 (47.0)	31 (36.5)	43 (51.2)	88 (50.3)	49 (36.6)	51 (41.5)	101 (59.1)
Immunocompromised *n* (%)							
Chemotherapy last 30 days	213 (27.6)	26 (30.2)	32 (38.1)	42 (24.0)	20 (14.9)	24 (19.5)	69 (40.4)
Systemic steroid treatment	150 (19.4)	26 (30.2)	27 (32.1)	47 (26.9)	8 (6.0)	12 (9.8)	30 (17.5)
Neutropenia	183 (23.7)	13 (15.1)	16 (19.0)	55 (31.4)	28 (20.9)	19 (15.4)	52 (30.4)
Solid organ transplantation	62 (8.0)	7 (8.1)	0 (0.0)	34 (19.4)	2 (1.5)	7 (5.7)	12 (7.0)
Bone marrow transplant	21 (2.7)	3 (3.5)	0 (0.0)	0 (0.0)	0 (0.0)	6 (4.9)	12 (7.0)
Chronic dialysis	36 (4.7)	4 (4.7)	5 (6.0)	6 (3.4)	4 (3.0)	8 (6.5)	9 (5.3)
Surgery last 30 days, *n* (%)	226 (29.5)	19 (22.4)	16 (19.0)	67 (38.3)	24 (18.6)	44 (36.1)	56 (32.7)
Department of hospitalization, *n* (%)							
Surgical	119 (15.4)	19 (22.1)	9 (10.7)	25 (14.3)	16 (11.9)	15 (12.3)	35 (20.5)
Medical	479 (62.0)	59 (68.6)	52 (61.9)	104 (59.4)	78 (58.2)	68 (55.7)	118 (69.0)
Intensive Care Unit	174 (22.5)	8 (9.3)	23 (27.4)	46 (26.3)	40 (29.9)	39 (32.0)	18 (10.5)
Arrival from, *n* (%)							
Home	656 (85.0)	75 (87.2)	74 (88.1)	156 (89.1)	117 (88.0)	92 (74.8)	142 (83.0)
Nursing home	55 (7.1)	9 (10.5)	2 (2.4)	6 (3.4)	10 (7.5)	11 (8.9)	17 (9.9)
Another hospital	61 (7.9)	2 (2.3)	8 (9.5)	13 (7.4)	6 (4.5)	20 (16.3)	12 (7.0)
Place of acquisition, *n* (%)							
Unknown	9 (1.2)	0 (0.0)	0 (0.0)	1 (0.6)	6 (4.5)	2 (1.6)	0 (0.0)
Community	100 (12.9)	16 (18.6)	6 (7.1)	15 (8.6)	29 (21.6)	26 (21.1)	8 (4.7)
Healthcare associated	254 (32.9)	25 (29.1)	24 (28.6)	55 (31.4)	37 (27.6)	18 (14.6)	95 (55.6)
Nosocomial	410 (53.0)	45 (52.3)	54 (64.3)	104 (59.4)	62 (46.3)	77 (62.6)	68 (39.8)
Length of stay, Median [IQR]	20.0 [9.0, 41.0]	20.0 [10.0, 43.5]	29.0 [17.5, 52.5]	25.0 [12.0, 44.0]	13.0 [7.0, 30.0]	24.5 [12.0, 53.8]	13.0 [6.0, 30.5]
Source of infection, *n* (%)							
Unknown	142 (18.4)	13 (15.1)	37 (44.0)	17 (9.7)	26 (19.4)	31 (25.2)	18 (10.5)
Abdominal	77 (10.0)	0 (0.0)	2 (2.4)	30 (17.1)	17 (12.7)	12 (9.8)	16 (9.4)
Vascular catheter	130 (16.8)	21 (24.4)	21 (25.0)	21 (12.0)	11 (8.2)	19 (15.4)	37 (21.6)
Pulmonary	159 (20.6)	11 (12.8)	14 (16.7)	53 (30.3)	28 (20.9)	26 (21.1)	27 (15.8)
Skin/soft tissue/bone/joint	73 (9.4)	3 (3.5)	4 (4.8)	20 (11.4)	8 (6.0)	18 (14.6)	20 (11.7)
Urinary	178 (23.0)	29 (33.7)	6 (7.1)	34 (19.4)	42 (31.3)	16 (13.0)	51 (29.8)
Other	14 (1.8)	9 (10.5)	0 (0.0)	0 (0.0)	2 (1.5)	1 (0.8)	2 (1.2)
Multidrug-resistant *P. aeruginosa*, *n* (%)	112 (14.5)	2 (2.3)	24 (28.6)	45 (25.7)	11 (8.2)	25 (20.3)	5 (2.9)
Extensively drug-resistant *P. aeruginosa*, *n* (%)	50 (6.5)	0 (0.0)	18 (21.4)	13 (7.4)	3 (2.2)	15 (12.2)	1 (0.6)
Appropriate empiric antibiotic treatment, *n* (%)	655 (84.7)	69 (80.2)	80 (95.2)	172 (98.3)	97 (72.4)	115 (93.5)	122 (71.3)
Septic shock at onset, *n* (%)	115 (18.0)	7 (8.1)	18 (21.4)	35 (20.0)	*NA*	31 (25.2)	24 (14.0)
Mortality day 7, *n* (%)	120 (15.5)	7 (8.1)	18 (21.4)	31 (17.7)	14 (10.4)	31 (25.2)	19 (11.1)
Mortality day 30, *n* (%)	182 (23.5)	9 (10.5)	23 (27.4)	47 (26.9)	25 (18.7)	51 (41.5)	27 (15.8)

*n c*ount, *IQR* interquartile Range.

### Distribution of sequence types, identification of virulence clusters, and association of bacterial factors with patient characteristics

**Fig. 3:**
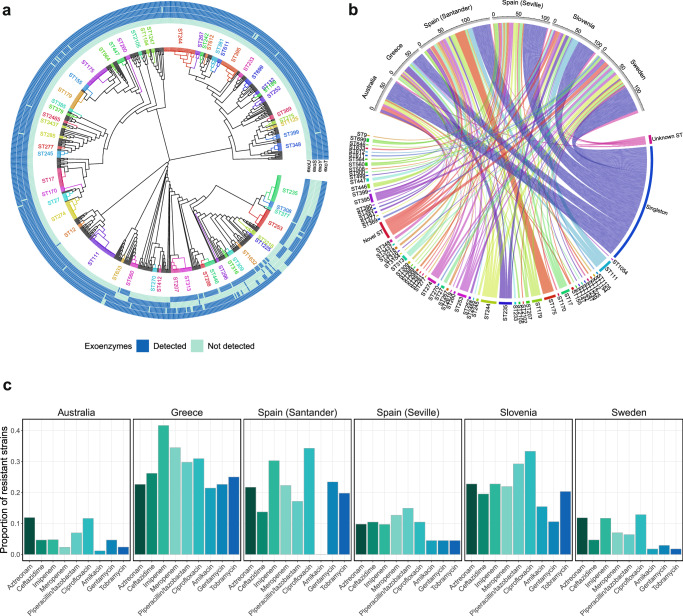
The evolutionary relationships and geographical distribution among *P. aeruginosa* bloodstream infection isolates. **a** Phylogenetic tree of the *P. aeruginosa* Multilocus Sequence Types (MLST) (*n* = 773). Tree color annotation is based on STs occurring more than twice in the dataset. The outer circles show a heatmap of exoenzyme gene detection (dark) or not (light). **b** Circle plot showing the geographical distribution of all the different STs from the 773 samples. The scale below the name of the study site marks the number of isolates. **c** Prevalence of antimicrobial resistance stratified by study site (Australia *n* = 86, Greece *n* = 84, Spain [Santander] *n* = 175, Spain [Seville] *n* = 134, Slovenia *n* = 123, and Sweden *n* = 171). Sequence Type (ST).

The phylogenetic tree of all sequenced isolates is presented in Fig. 3a. The most frequently occurring sequence type was ST244, with 36 cases, followed by ST111 with 34, ST235 with 31, and ST175 with 28. In total 142 of 773 (18.3%) STs occurred only once, 41 of 773 (5.3%) were new STs and 21 of 773 (2.7%) were unknown STs. The geographical distribution of antimicrobial resistance and STs is shown in Fig. 3b-c. Notably, ST244 was present in all locations, ST111 was present in Greece, Spain (Santander and Seville), and Slovenia, ST235 was present in Greece, Spain (Santander and Seville), Slovenia, and Sweden, and ST175 was present in Spain (Santander), Slovenia and Sweden. The prevalence of the MDR phenotype varied significantly by sequence type (p = 0.0001), with 85.3% of ST111 isolates, 71.4% of ST175 isolates, and 64.5% of ST235 isolates displaying such resistance (Supplementary Table 4). Epidemic clones were categorized as STs occurring more than 10 times in the cohort (n = 11 different STs). In total, 83 out of 112 MDR isolates (74.1%) were part of one of the epidemic clones. The *exoU* genotype was prevalent in ST235 (31/31 isolates), ST253 (22/22 isolates), ST313 (14/14 isolates), and ST446 (10/12 isolates) but was not present in any of the other epidemic clones (Fig. 3a). There was a relatively even distribution of sources of infection among the different STs, except for ST175, where pulmonary infections were common, and ST313, where urinary infections were common (Supplementary Fig. 3).

**Fig. 4:**
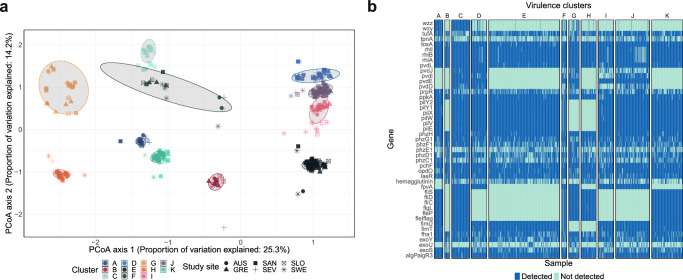
Identification of genotypic virulence clusters. **a** Principal Coordinate Analysis based on the virulence gene content shown in two dimensions. Cluster color annotation has been calculated using k-means clustering. The ellipse covers 95% of the isolates in each cluster. **b** Heatmap of the virulence genes with the most variability (*n* = 45) on the y-axis and samples (*n* = 773) on the x-axis. All isolates were annotated based on virulence gene detection (dark) or not (light) and stratified by virulence cluster A to K (each cluster separated by a white space). Principal Coordinate Analysis (PCoA), Australia (AUS), Greece (GRE), Spain Santander (SAN), Spain Seville (SEV), Slovenia (SLO), and Sweden (SWE).

After conducting Principal Coordinate Analysis (PCoA) based on a matrix of all 773 samples with annotated virulence genes from the VFDB reference database (*n* = 247), 11 genotypic virulence clusters (Clusters A–K) were identified using an unsupervised k-means clustering algorithm (Fig. 4a). Each cluster was represented by a distinct virulence genotype and were present in several geographical locations (Fig. 4a, b). The clusters differed in size ranging between 18 and 237 samples and were dominated by different STs, of which the most prominent were: cluster A by ST447 (9/17, 53%) and ST245 (8/17, 47%), cluster C by ST244 (36/48, 75%), cluster D by ST348 (6/20, 30%), cluster E by ST235 (31/90, 34%), ST179 (23/90, 26%) and ST17 (21/90, 23%), cluster G by ST253 (22/29, 76%), cluster H by ST274 (22/28, 79%), cluster I by ST446 (12/27, 44%), cluster J by ST175 (22/69, 32%) and ST395 (14/69, 20%), and cluster K by ST111 (34/43, 79%) (Supplementary Fig. 4 and Supplementary Table 5). Two smaller clusters (clusters B and F) were not dominated by any of the common STs.

**Fig. 5:**
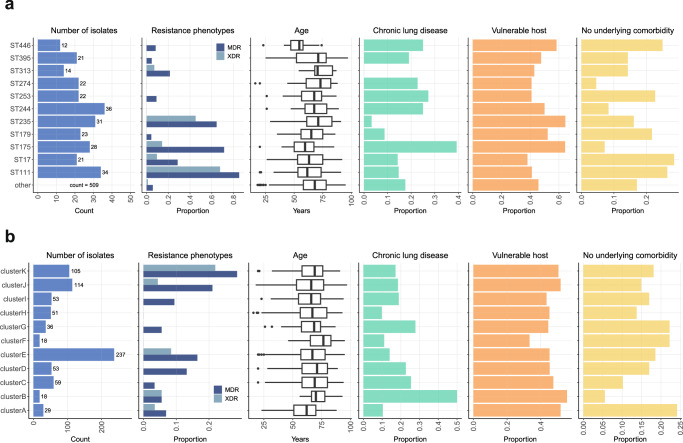
Relationship between bacterial genotype, multidrug resistance, and patient characteristics. The figure is organized from left to right, starting with the number of strains in each group displayed in the first bar chart. In the subsequent graphs to the right, the proportions and the box plot are derived from these groups. **a** Distribution of resistance phenotypes, age, chronic lung disease, vulnerable host characteristics, and no underlying comorbidity in sequence types occurring >10 times in the cohort. **b** Distribution of resistance phenotypes, age, chronic lung disease, vulnerable hosts characteristics, and no underlying comorbidity in the virulence cluster. In both (**a** and **b**), Chronic lung disease was defined according to the Charlson Comorbidity Index, and vulnerable host characteristics were defined as either chemotherapy during the last 30 days, systemic corticosteroid treatment (>10 milligrams of prednisone for >29 days), neutropenia (absolute neutrophil count <0.5 × 109/liter), solid organ transplant, bone marrow transplant and/or chronic dialysis treatment. The age distribution is displayed in a box plot showing the median, interquartile range [IQR], whiskers extending 1.5 * IQR, and outliers. Multidrug-resistant (MDR), Extensively drug-resistant (XDR).

Based on the hypothesis that the invasive disease potential of PA isolates was affected by patient characteristics, we evaluated the relationship between patient characteristics and genomic markers of virulence. Using prior knowledge, three important patient groups were identified: (1) hosts with chronic lung disease, (2) vulnerable hosts, defined as immunocompromised patients, and (3) hosts with no underlying comorbidity, defined as a CCI of zero point. The relationship between epidemic clones, age, and comorbidities is shown in Fig. 5a. There was a significant variation in patient age depending on the epidemic clone involved (*p* = 0.009). Patients infected with ST175 had a median age of 60 years (IQR 50–67), and those with ST446 had a median age of 55 years (IQR 47–58), both representing younger age groups compared to the overall cohort. In contrast, patients with ST313 were older, with a median age of 71 years (IQR 68–84 years). Chronic lung disease was particularly prevalent among ST175, affecting 11 of 28 patients (39%), as opposed to ST235 where only 1 of 31 patients (3%) had these conditions (*p* = 0.01). None of the 14 patients infected with ST313 exhibited chronic lung disease. There was no significant association between epidemic clones and either vulnerable hosts or the absence of underlying comorbidities (*p* = 0.50 and 0.24, respectively). The proportion of MDR isolates, as well as the proportion of chronic lung disease, differed between the virulence clusters A–K (*p* = 0.0001 and *p* = 0.01, respectively). Age, immunosuppression, or no registered comorbidities were not significantly different according to cluster groups (Fig. 5b). In addition, the T3SS genotype was associated with an MDR phenotype, but not with age or comorbidities (Supplementary Table 4).

### The virulence genotype impacts patient outcomes

**Fig. 6:**
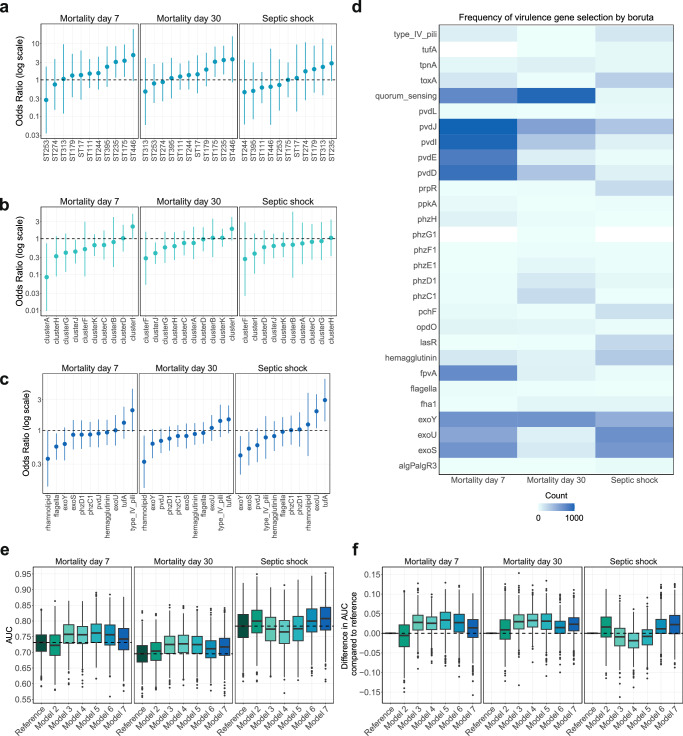
Association between genomic virulence markers and patient outcomes. The sample size was *n* = 772 due to missing data on the department of hospitalization for 1 patient. **a–c** Logistic regression models fitted for virulence factors with *p*-value < 0.1 in the univariate analysis, epidemic clones with all other STs as reference, and virulence cluster with the largest cluster E as reference. All models were adjusted for confounders: geographical site, age group, sex, Charlson comorbidity index group, immunosuppressed state, department of hospitalization, and nosocomial infection. The dashed line marks odds ratio = 1, the circles symbolize the odds ratios for each predictor variable, and the error bars show the 95% confidence interval for each odds ratio. **d** The importance of specific virulence genes to predict outcomes based on the Boruta feature selection algorithm applied in the development set for each of the 1000 random splits of the data into the development and validation set. Genes selected at least one time are shown on the y-axis. The heatmap color is based on the number of times a feature was selected ranging between 1 and 1000, with lighter colors indicating lower counts and darker colors indicating higher counts. **e** Box plot (median, interquartile range [IQR], whiskers extending 1.5 * IQR, and outliers) of 6 random forest classifiers fitted to predict mortality or septic shock and assessed in the validation set. All models were based on different combinations of predictors. *Reference model*: patient characteristics alone (age, sex, comorbidity, immunosuppression, hospital department, and nosocomial infection), *Model 2*: combination of patient characteristics and all filtered and grouped virulence genes (*n* = 29), *Model 3*: combination of patient characteristics and epidemic clones occurring >5 times (*n* = 25), *Model 4*: combination of patient characteristics and epidemic clones occurring >10 times (*n* = 11), *Model 5*: combination of patient characteristics and virulence clusters A–K, *Model 6*: combination of patient characteristics and virulence genes selected by the Boruta algorithm, and *Model 7*: combination of patient characteristics with virulence genes and resistance genes selected by the Boruta algorithm. The plot shows the area under receiver operating characteristics (AUROC) for each of the 1000 random splits of the data into development and validation set, with a dashed line marking the median of the Reference model. **f** Box plot (median, IQR, whiskers extending 1.5 * IQR, and outliers) of the difference in AUROC between Models 2–7 and the Reference model for each of the 1000 splits, with a dashed line marking 0.00 difference. A positive difference occurs when the AUROC of the virulence model is greater than the Reference model. A negative difference occurs when the AUROC of the virulence model is less than the Reference model.

The association between genomic virulence markers and patient outcomes was evaluated through three different data-driven approaches to define exposure. These approaches varied in how they aggregated virulence genotype data: (1) based on prevalent bacterial clones, (2) based on identifiable clusters of virulence genes, and (3) based on individual virulence genes (Fig. 6). The association of virulence genomic markers with patient outcomes based on univariable logistic regression is presented in Supplementary Tables 6–8. In the adjusted multivariable model, both ST235 and ST175 were significantly associated with higher 7-day and 30-day mortality rates after accounting for patient-related confounders (Fig. 6a and Supplementary Tables 9–14). For ST235, the odds ratio (OR) for 7-day mortality was 3.12 (95% CI: 1.15–8.45, *p* = 0.03), and for ST175, the OR was 3.38 (95% CI: 1.21–9.43, *p* = 0.02). For 30-day mortality, ST235 had an OR of 3.51 (95% CI: 1.42–8.68, *p* = 0.01), while ST175 had an OR of 3.16 (95% CI: 1.21–8.26, *p* = 0.02). Additionally, ST446 showed a trend toward increased mortality, with an OR of 4.82 (95% CI: 0.92–25.14, *p* = 0.06) for 7-day mortality and an OR of 3.69 (95% CI: 0.86–15.81, *p* = 0.08) for 30-day mortality. While no specific sequence types were statistically associated with the occurrence of septic shock at the onset of bloodstream infection, a notable trend was observed for ST235 and ST179, both belonging to virulence cluster E, and ST313 distributed in several clusters. Since MDR phenotype was common in ST235 and ST175, it was unclear if these findings were due to virulence or inappropriate antimicrobial treatment. Based on this, we performed a post hoc analysis also adjusting for receiving appropriate empiric antibiotic treatment. The analysis had limited impact on the results and there was still an increased 7-day mortality (ST235 OR 3.18 [95% CI, 1.17–8.66], *p* = 0.02; and ST175 OR 3.36 [95% CI, 1.20–9.45], *p* = 0.02) and 30-day mortality (ST235 OR 3.54 [95% CI, 1.43–8.77], *p* = 0.006; and ST175 OR 3.16 [95% CI, 1.20–8.25], *p* = 0.02), but no increased risk of septic shock (ST235 OR 2.87 [95% CI, 0.93–8.89], *p* = 0.07; and ST175 OR 1.02 [95% CI, 0.34–3.06], *p* = 0.97). In the analyses adjusting for appropriate treatment, ST446 was now significantly associated with 7-day mortality (OR 5.29 [95% CI, 1.02–27.48], *p* = 0.05), but not with 30-day mortality (OR 3.78 [95% CI, 0.88-16.18], *p* = 0.07) or septic shock (OR 0.64 [95% CI, 0.06–7.13], *p* = 0.71).

Compared to the largest virulence cluster E (reference), clusters A and J were associated with a decreased risk of 7-day mortality, while only cluster J was associated with a decreased risk of 30-day mortality (Fig. 6b and Supplementary Tables 9–14). In the multivariable analysis, none of the virulence clusters correlated with septic shock. These findings were consistent also after adjusting for appropriate empirical treatment for 7-day mortality (cluster A, OR 0.07 [95% CI, 0.01–0.67], *p* = 0.02; and cluster J, OR 0.44 [95% CI, 0.21–0.95], *p* = 0.04), 30-day mortality (cluster J, OR 0.40 [95% CI, 0.20–0.79], *p* = 0.01) and septic shock (cluster A, OR 0.76 [95% CI, 0.20–2.87], *p* = 0.68; and cluster J, OR 0.63 [95% CI, 0.29–1.37], *p* = 0.24).

When assessing individual virulence genes, homology with the reference protein of genes coding for flagella function (*flaG, fleP, flg, fliC, fliD, fliS*) and quorum sensing (*rhlI, rhlA, rhlB*) showed a negative association with 7-day mortality, with OR 0.56 (95% CI, 0.35–0.90), *p* = 0.02; and OR 0.36 (95% CI, 0.13–0.98), *p* = 0.05, respectively (Fig. 6c). For 30-day mortality, the odds ratios had a similar distribution, but the associations were weaker. Detection of the *exoU* genotype and Elongation factor Tu (*tufA*) was linked to septic shock, with OR 1.99 (95% CI, 1.08–3.64), *p* = 0.03; and OR 2.96 (95% CI, 1.38–6.34), *p* = 0.005, respectively. However, these factors were not associated with mortality. Conversely, the *exoS* and *exoY* genotypes showed a negative association with septic shock, yielding OR 0.52 [95% CI, 0.29–0.95], *p* = 0.03; and OR 0.41 [95% CI, 0.21–0.81], *p* = 0.01, respectively.

### Virulence gene markers add prognostic information to a machine learning model

To evaluate the prognostic importance of virulence genotype on patient outcomes, we developed and tested multiple predictive models using a random forest classifier. The models were based on a different set of variables: (Reference model) patient characteristics alone (age, sex, comorbidity, nosocomial infection, hospital department, immunosuppression), (Model 2) combination of patient characteristics and all filtered and grouped virulence genes (*n* = 29), (Model 3) combination of patient characteristics and epidemic clones occurring >5 times (*n* = 25), (Model 4) combination of patient characteristics and epidemic clones occurring >10 times (*n* = 11), (Model 5) combination of patient characteristics and virulence clusters A–K, (Model 6) combination of patient factors and virulence genes selected by the Boruta feature selection algorithm and (Model 7) combination of patient factors with virulence and resistance genes selected by the Boruta algorithm. During the training and testing process, the cohort was split randomly 80/20 into a development and validation set. To account for data split variability, this procedure was repeated 1000 times using distinct random seeds and the predictive performances are presented as the distribution of the AUROC for each model (Fig. 6e).

In prediction of 7-day mortality, the median AUROC was 0.73 (IQR 0.70–0.76) for the Reference model based on patient characteristics alone, compared to the models also using information on virulence genotype, as follows: Model 2, 0.72 (IQR 0.69–0.76); Model 3, 0.76 (IQR 0.73–0.79); Model 4, 0.76 (IQR 0.73–0.78); Model 5, 0.76 (IQR 0.73–0.79); Model 6, 0.76 (IQR 0.72–0.79); and Model 7, 0.74 (IQR 0.71–0.78). A similar pattern was seen in the prediction of 30-day mortality, with median AUROC 0.70 (IQR 0.67–0.72) for the Reference model, compared to Model 2, 0.70 (IQR 0.67–0.73); Model 3, 0.73 (IQR 0.70–0.75); Model 4, 0.73 (IQR 0.70–0.75); Model 5, 0.72 (IQR 0.70–0.75); Model 6, 0.71 (IQR 0.68–0.74); and Model 7, 0.72 (IQR 0.69–0.74). In septic shock, median AUROC for the Reference model was 0.78 (IQR 0.75–0.82), compared to Model 2, 0.80 (IQR 0.76–0.83); Model 3, 0.77 (IQR 0.74–0.81); Model 4, 0.77 (IQR 0.73–0.80); Model 5, 0.78 (IQR 0.74–0.81); Model 6, 0.80 (IQR 0.76–0.84); and Model 7, 0.81 (IQR 0.77–0.84).

In addition to assessing the median AUROC, the difference in AUROC between models that included virulence data and the reference model was calculated for each of the 1000 splits. A positive difference implies that the AUROC of the virulence model is greater than the reference model, and a negative difference implies that the AUROC of the virulence model is less than the reference model. For mortality prediction, the differences in AUROC between Models 3–7 and the reference model were generally skewed toward positive values, except for Model 7. Notably, these models had higher AUROC than the reference model in 75% of the 1000 algorithm runs (Fig. 6f). For the prediction of septic shock, the model including genes selected by the Boruta algorithm (Models 6 and 7) performed better than the reference model in 75% algorithm runs, but none of the other models improved prediction compared to using only patient characteristics. For all outcomes, Model 2, which included all virulence genes, generally performed similarly to the Reference model, indicating that just adding more data was not the reason for improved predictive performance. A sensitivity analysis using a stricter gene identity threshold of 95%, allowing for less gene variation, yielded similar results to the main analysis with a gene identity threshold of 80% (Supplementary Fig. 5).

In model 6, the importance of specific virulence genes to predict outcome was assessed with the Boruta feature selection algorithm which is also based on a random forest model. These variables were extracted and presented in a heatmap based on how often they were selected across the 1000 random development datasets (Fig. 6d). For the prediction of 7-day mortality, the following variables were most frequently identified as important by the algorithm: pyoverdine-related (*fpvA, pvdD, pvdI, pvdJ, pvdE*), quorum sensing (*rhlA, rhlB*, *rhlI*), and T3SS (*exoU*, *exoS*, *exoY*). For the prediction of 30-day mortality, the algorithm most frequently selected quorum sensing, *exoY*, *pvdJ, pvdD*, and *pvdI* as important variables. In the case of septic shock, the T3SS-related effector proteins *exoU, exoS*, and *exoY*, along with *pvdJ* and hemagglutinin, were most often chosen by the algorithm. The Boruta feature selection results, considering both virulence and resistance genes, are presented in Supplementary Fig. 6.

## Discussion

In this whole-genome sequencing study of 773 PA isolates from clinical bloodstream infections, we observed associations between genomic markers of bacterial virulence and severe patient outcomes. Based on the bacterial genotype we identified several virulence clusters characterized by specific sequence types, each showing varying proportions of MDR phenotypes. Infections involving the high-risk clones, ST175 and ST235, were associated with higher mortality, even after adjusting for host factors and empirical treatment. Taken together, these findings suggest that genomic markers provide prognostic information in severe PA infections, illustrating how bacterial WGS data may impact diagnostic accuracy in sepsis.

What sets our study apart is its scale and collection of bacterial strains from all instances of PA BSI, rather than being limited to selected cases. Clinical and bacterial data was also gathered in a highly standardized format from multiple geographical locations in Europe and Australia, supporting the generalizability of our results across different settings and regions. An important aspect of bacterial infections and vaccine development is the invasive disease potential of clones. Although PA generally exhibits a non-clonal population structure, we observed that one-third of bloodstream infections were attributable to one of the 11 most common clones. Notably, these clones were responsible for 75% of MDR phenotypes. Previous data have reported widespread global transmission of ST235, ST111, and ST244, but also ST175 primarily in Europe^9,51^. In other pathogens, such as invasive pneumococcal disease, molecular epidemiological studies suggest that certain clones are more likely to affect younger, healthier patients, while clones with lower invasive potential tend to primarily affect vulnerable hosts^52^. In this study, patients infected with ST175 and ST446 were generally younger and had high proportions of chronic lung disease. In addition, the pulmonary source of BSI was dominating in infections caused by ST175, suggesting a general affinity to airways. On the contrary, the *exoU*-positive strains ST235 and ST313 occurred frequently in older patients but were mostly absent in patients with chronic lung disease. Approximately one fourth of patients with ST17, ST111, ST446, ST179, and ST253 did not have any underlying comorbidities, however, there was no statistically significant association between previous healthy patients and epidemic clones. When analyzing the T3SS genotype, there was no major difference in patient characteristics. Collectively, these findings indicated that epidemic clones caused an important difference in the disease burden but did not provide clear evidence of differences in invasive disease potential between clones in PA BSI.

Based on PCoA of all annotated virulence genes, we identified 11 predominant virulence clusters. These clusters were linked with specific STs and exhibited varying proportions of MDR phenotypes, well in line with previous findings of an association between clonal complex and virulence phenotype^12,53,54^. Some of the STs and virulence clusters were associated with mortality in the multivariable logistic regression analyses. Additionally, machine learning models using both patient and virulence cluster or epidemic clone data generally performed better than models using only patient characteristics in predicting mortality. For predicting septic shock, the inclusion of data on epidemic clones or virulence clusters did not enhance the model’s predictive power. This finding that both ST and virulence clusters were associated with mortality, but not clearly with septic shock may have several explanations. First, although we tried to minimize bias by adjusting for important factors such as age, comorbidity, and department, unmeasured confounding may still be present. Second, while this is one of the largest cohorts of consecutively collected PA BSI reported to date, the statistical power was insufficient to detect minor increases in the risk of outcomes, particularly in the case of septic shock, where data was absent from one study site. Third, the classification of septic shock followed the established Sepsis-3 clinical criteria and required patients to receive vasopressor support^55^. This might exclude patients with care limitations who may still meet the pathophysiological definition of septic shock. Consequently, the mortality data may provide a more robust measure of disease severity.

In the multivariable logistic regression models of individual gene markers, we predominantly identified factors with a negative association with mortality, such as genes tied to flagella function or quorum sensing. This indicates species traits that may alleviate bacterial pathogenicity in invasive PA disease. Flagella have previously been associated with immune activation and faster bacterial clearance in mice, which may affect virulence also in human infection^56^. Quorum sensing is crucial for sustaining biofilm integrity, and it is often linked to chronic infections. In such settings, host adaptations tend to be more finely calibrated and virulence is generally less pronounced^57,58^. In septic shock, we mainly found an association with T3SS. When activated, the T3SS secretes one of the effector proteins—*exoS, exoT, exoU*, or *exoY*—facilitating the invasion of epithelial cells and triggering an immune response^59^. Previous studies have shown the prevalence of *exoS* (poly-substrate toxin) in approximately 70–80% and *exoU* (phospholipase) in approximately 20–30% of isolates from clinical infections, but rarely both in the same isolate, which was also the case in our cohort^59,60^. PA strains known to secrete any of the T3SS effector proteins, primarily *exoU*, have been linked to poorer clinical outcomes in both ventilator-associated pneumonia and BSI^17,18^. The *exoU* genotype has also been associated with increased exacerbations in bronchiectasis patients^61^. A study by Peña et al. examined a large cohort of Spanish PA BSI cases and found that the *exoU* genotype was correlated with mortality within 5 days, but not 30-day mortality^24^. In this study we observed an association between *exoU* and septic shock at onset, indicating a clinically relevant impact of T3SS on patient outcome. However, we could not replicate the findings on mortality from the study by Peña et al., which may be due to differences in clinical management, or that the association between *exoU* and early death is either weak or absent^24^. Similar findings were reported by a research team examining the same cohort as Peña et al. ^24^. This group further categorized the PA isolates based on their virulence phenotype using a *Caenorhabditis elegans* infection model. Although they found a strong correlation between the virulence phenotype and the T3SS genotype, the model turned out to be a poor predictor of mortality in bloodstream infections^62^. The investigators examined virulence phenotypes at a general level, whereas other studies have focused on specific virulence factors. For example, Zupetic et al. assessed elastase expression and found that increased activity was associated with higher mortality in ICU patients^23^. In a mouse model, they also demonstrated that elevated elastase production led to greater lung tissue damage. This underscores the complexity of determining bacterial virulence in vivo, suggesting that future analyses may need to focus on the expression of specific virulence traits and functional virulence assays.

We also found a negative association between *exoS* and *exoY* genotypes with septic shock. Since *exoS* and *exoU* rarely co-exist, our finding is unsurprising. However, a recent population genomic study suggested that this may indicate different subspecies, potentially contributing to other differences in virulence beyond those explained by the T3SS^60^. The role of *exoY* in human infection has not been as well characterized as *exoU*. An experimental study showed that approximately 60% of isolates harboring the *exoY* gene actually secreted the toxin^63^. The same study also noted that secreted *exoY* counteracted the overall cytotoxicity of PA and possibly had a protective effect to facilitate host colonization and maintain chronic infection, which may explain our findings^63^. Interestingly, both *exoS*, *exoT* and *exoY* have been shown to attenuate the cytotoxic activity of *exoU*, especially during chronic infection^64^. Several of our isolates carried both the *exoY* and *exoU* genotype and we cannot exclude a complex phenotypic interaction in vivo between these effectors. Furthermore, the *tufA* genotype was also associated with septic shock, but the mechanism behind this finding is unclear. The *tufA* gene is an important component of protein synthesis mainly located in the cytoplasmic space, but studies have also observed thermoregulated *tufA* gene expression on the cell-surface in PA with implications for bacterial adherence^65,66^.

We have previously reported several patient related, and mainly unmodifiable, factors associated with mortality in PA BSI such as age, sex, functional capacity, recent hospitalization, concomitant corticosteroids, CCI, hospital-acquired infection, endotracheal tube at baseline and admission to ICU^6^. In this study, we also wanted to evaluate the added prognostic value of virulence genotype data. With WGS now economically viable for routine diagnostics in clinical microbiological laboratories, molecular characterization can be completed within hours, enabling timely decisions about clones and virulence genes. This capacity for rapid data extraction can directly impact patient care, improving diagnostic accuracy and guiding tailored treatment strategies. The utility of using genotype for predicting pathogenicity in PA was recently challenged by Panayidou et al., who argued that variations in virulence from strain to strain cannot be accurately determined at the genome level^67^. Instead, they suggested that this should be assessed at the pathway level through functional transcriptomics^67^. Conversely, Pincus et al. demonstrated that a genome-based machine learning model was effective in predicting PA virulence across 115 clinical isolates, primarily from BSI^35^. However, this prediction was possible through a diffuse genomic signature rather than relying on individual genes^35^. Our focus was rather to predict clinically relevant patient outcomes, and we assessed several different levels of aggregation of virulence genotype. To account for complex nonlinear interactions between predictors, we chose a random forest model. We observed a variability in algorithm performance based on the resampling of the random 80/20 split, which may be related to the sample size of the cohort. Despite these limitations, the models incorporating selected virulence data did perform better than a model relying solely on patient characteristics, but the difference in AUROC between models was small. From a clinical standpoint, the priority of collecting virulence information in acute PA infection to forecast patient outcomes can be debated, especially since WGS requires additional resources. On the other hand, our findings indicate that virulence genomic markers contain prognostic information that may inform clinical decisions.

Our study has limitations. Although it is one of the largest cohorts of consecutively collected PA BSI reported to date, statistical power was insufficient to assess rare virulence genotypes or bacterial clones in a meaningful way. The study was pragmatically designed to evaluate readily accessible virulence data for integration into a clinical analytical pipeline. Consequently, we focused on previously described virulence genes from publicly available databases and did not account for other genomic factors, such as specific mutations or transcriptomics. This approach comes with the caveat that we did not evaluate the virulence phenotype, and it remains uncertain whether our genotype classifications consistently correspond to actual virulence traits across all isolates. As an example, it has been shown that mutations introduced into the needle protein PscF may abrogate the function of the T3SS^68^. On the other hand, prior lab studies in PA have established a clear association between the virulence genotype and the T3SS virulence phenotype^32^. We did assess the impact of genetic variation by adjusting gene identity thresholds, but this did not substantially alter our findings.

The study period spanned over 7 years, and it is possible that the properties of different STs may have evolved over time due to changes in integrons, as well as other horizontal gene transfers or chromosomal gene mutations, possibly affecting the associations between ST and patient outcome. As these factors continue to evolve in the future, the applicability of our results may also change. Our analyses of STs could also be affected by geographical bias since we might have overlooked important clones present in other settings not included in this study. To overcome some of these issues, we did not only look at STs but also studied the individual virulence genotype. Furthermore, the associations between virulence genomic markers and patient outcomes showed varying degrees of statistical certainty. Since we used an exploratory approach and performed multiple tests, it is possible that some of the associations can be attributed to chance rather than a true association. However, our findings on T3SS-mediated virulence align with previous smaller clinical and experimental studies, supporting their relevance^17,18,24^. In an effort to understand the influence of PA virulence genotype on mortality rates and the onset of septic shock, we trained and assessed machine learning models using different clinical and bacterial data. Even though we used a recognized method of internal validation, these results are not sufficient for prediction in a clinical setting, which would require confirmation in external datasets to rule out model overfitting.

In conclusion, we found associations between bacterial virulence genotypes and adverse clinical outcomes such as increased mortality and septic shock in PA BSI. Specifically, infections with epidemic clones ST175 and ST235 were linked to higher mortality rates, even after controlling for host factors and empirical treatment. Incorporating virulence genotype information into predictive models based on patient characteristics indicated an improvement in the prediction of severe outcomes in PA BSI patients. These findings imply that bacterial genomic markers offer prognostic insights in severe PA infections and may guide adjuvant sepsis treatments or vaccine development, although further validation is needed.

## Supplementary information

Supplementary Information

Reporting Summary

## Data Availability

The collected *P. aeruginosa* strains in this study are available from the corresponding author on reasonable request with a completed materials transfer agreement. The genomes of all sequenced bacterial strains are deposited in the Sequence Read Archive (SRA), maintained by The National Center for Biotechnology Information (NCBI) under the accession number PRJNA1189087. A dataset featuring annotated virulence genome data, resistance phenotype data, and the source data of the main figures is publicly available at https://data.mendeley.com/datasets/3h9gvbzz7x/2. According to current regulations, sharing restrictions apply to the dataset containing clinical parameters to safeguard the confidentiality and integrity of the study participants. Full access to patient-level data will require obtaining an ethical permit from local ethical review boards, as well as entering into a formal data-sharing agreement with the study investigators and/or affiliated institutions. For inquiries related to data access for this study, please contact the corresponding author. The study protocol is available from the corresponding author upon reasonable request.

## Code availability

The source code to generate the study’s main results is publicly available at https://data.mendeley.com/datasets/3h9gvbzz7x/2. The code is available under the license CC BY NC 3.0, meaning you are free to adapt, copy, or redistribute the material, providing you attribute appropriately and do not use the material for commercial purposes. All software and versions used in this study are listed in Supplementary Table 2.
