# Supplementary material for: Genomic virulence markers are associated with severe outcomes in patients with Pseudomonas aeruginosa bloodstream infection
Source: Commun Med (Lond). 2024 Dec 11;4:264. doi: 10.1038/s43856-024-00696-4 (PMC11634891; doi:10.1038/s43856-024-00696-4)
Supplement: Supplementary file 1 — Supplementary Information [file 43856_2024_696_MOESM1_ESM.pdf]

Supplementary Information

Genomic virulence markers are associated with severe outcomes in patients with *Pseudomonas aeruginosa* bloodstream infection

John Karlsson Valik*, Christian G. Giske, Badrul Hasan, Mónica Gozalo Margüello, Luis Martínez-Martínez, Manica Mueller Premru, Žiga Martinčič, Bojana Beović, Sofia Maraki, Maria Zacharioudaki, Diamantis Kofteridis, Kate McCarthy, David Paterson, Marina de Cueto, Isabel Morales, Leonard Leibovici, Tanya Babich, Fredrik Granath, Jesús Rodríguez-Baño, Antonio Oliver, Dafna Yahav and Pontus Nauclér.

***Corresponding author:** John Karlsson Valik, Department of Medicine, Solna, Division of Infectious Diseases, Karolinska Institutet, Stockholm, Sweden; tel. 0046 709 42 80 72; john.karlsson.valik@ki.se

Table of Content

**Supplementary Figure 1.** Elbow method to determine the number of K clusters	Page 3
**Supplementary Figure 2.** Directed acyclic graph	Page 4
**Supplementary Figure 3.** Source of infection stratified based on sequence type	Page 5
**Supplementary Figure 4.** Relationship between virulence clusters and sequence type	Page 6
**Supplementary Figure 5.** Impact of genetic variation on the predictive machine learning models	Page 7
**Supplementary Figure 6.** Boruta feature selection output when including both virulence and resistance genes	Page 8
**Supplementary Table 1.** Time period when the study sites contributed with data	Page 9
**Supplementary Table 2.** Material and software resources	Page 10
**Supplementary Table 3.** Whole genome sequence quality control	Page 11
**Supplementary Table 4.** Relationship between bacterial genotype, multidrug-resistance, and patient characteristics	Page 12
**Supplementary Table 5.** Relationship between virulence clusters and sequence type in *P. aeruginosa*	Page 13
**Supplementary Table 6.** Univariable logistic regression: 7-day mortality	Page 14
**Supplementary Table 7.** Univariable logistic regression: 30-day mortality	Page 15
**Supplementary Table 8.** Univariable logistic regression: septic shock	Page 16
**Supplementary Table 9.** Multivariable logistic regression: 7-day mortality	Page 17
**Supplementary Table 10.** Multivariable logistic regression: 30-day mortality	Page 18
**Supplementary Table 11.** Multivariable logistic regression: septic shock	Page 19
**Supplementary Table 12.** Multivariable logistic regression adjusted for appropriate empiric antibiotic treatment: 7-day mortality	Page 20
**Supplementary Table 13.** Multivariable logistic regression adjusted for appropriate empiric antibiotic treatment: 30-day mortality	Page 21
**Supplementary Table 14.** Multivariable logistic regression adjusted for appropriate empiric antibiotic treatment: septic shock	Page 22
**References**	Page 23

Supplementary Figure 1. Elbow method to determine the number of K clusters

The elbow method was used to determine the optimal number of clusters (k) in the cohort for the k-means clustering algorithm. A matrix of all samples (n=773) annotated against the Virulence Factor Database (VFDB) (n=247 genes) were used as data input. The algorithm was run n=100 times for a range of k values from 2 to 40 and the mean within-cluster-sum of squared errors (WCSS) for all runs are presented on the y-axis. The elbow point was determined by creating a line connecting the first and last mean WCSS points and then calculating the vertical distance between this line and the actual WCSS points for each k. The k value where the distance was maximum (k = 11) was considered the elbow point (red dot).

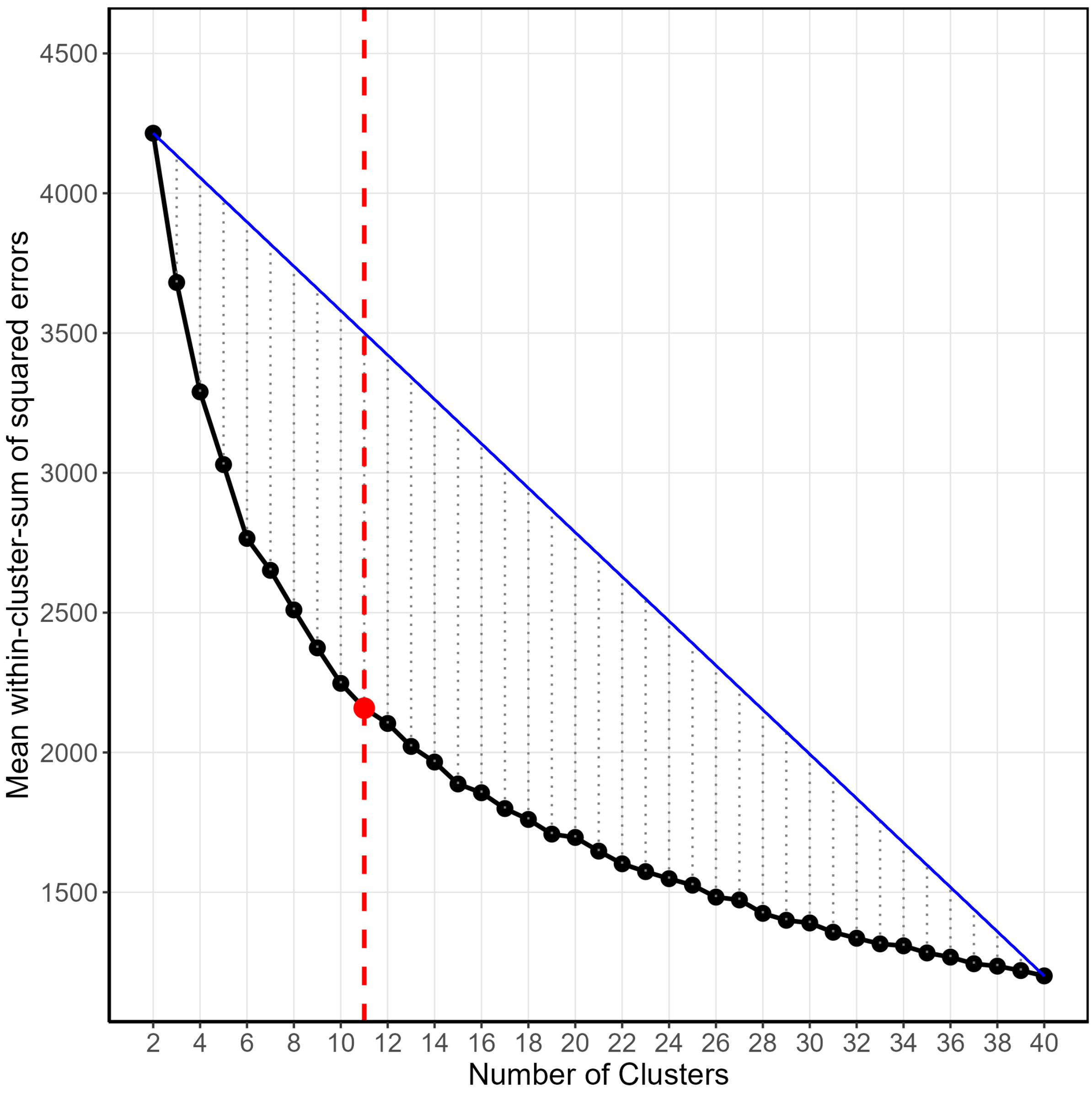

Supplementary Figure 2. Directed acyclic graph

A casual diagram was created to assess the relationship between patient variables and virulence genotype. The following variables were considered: main exposure=virulence factor, outcome=mortality (or septic shock at onset), Charlson Index, immunosuppression, age, intensive care unit (ICU) admission after onset, ICU care at onset, bacteremia onset (nosocomial vs community), antibiotic resistance, source of bacteremia, treatment, and health care system incl. the proxy for this, i.e., country. As can be seen from the figure antibiotic resistance, source of bacteremia, ICU admission after onset and treatment are classified as intermediate variables (blue dots) and should not be matched or adjusted for due to risk of inserting bias. Accordingly, possible confounders are: age, immunosuppression, Charlson index, country, ICU care at onset and nosocomial or community onset (red dots).

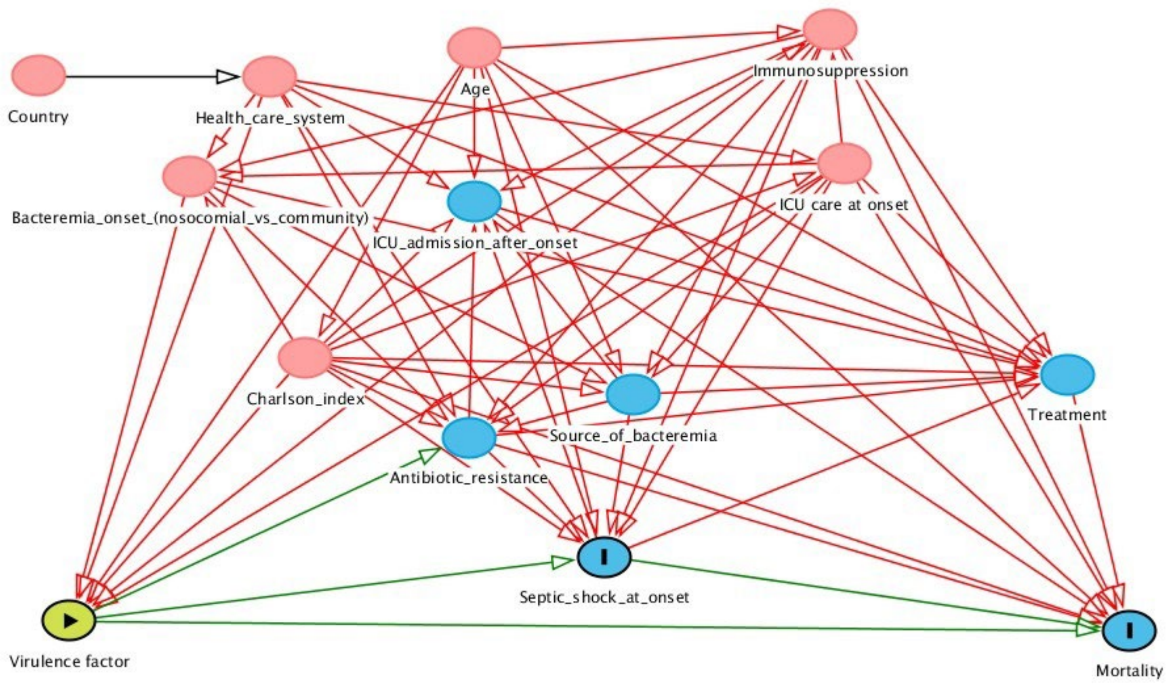

The DAG was created using the website www.dagitty.net (Textor, J., Hardt, J. & Knüppel, S. DAGitty: a graphical tool for analyzing causal diagrams. *Epidemiology* **22,** 745 (2011)

Supplementary Figure 3. Source of infection stratified based on sequence type

The distribution of source of infection for *Pseudomonas aeruginosa* bloodstream infections was plotted for each sequence type (ST). Only STs occurring more than ten times in the data set was mapped. The figure shows proportion of infection source on the Y-axis and STs on the X-axis. The X-axis is ranked from left to right according to frequency of STs in the entire data set (higher frequency to the left). The plot shows a relatively even distribution of sources of infection among the different STs, except for sequence type 175 which had pulmonary infections as dominating source and sequence type 313 which had urinary infections as dominating source.

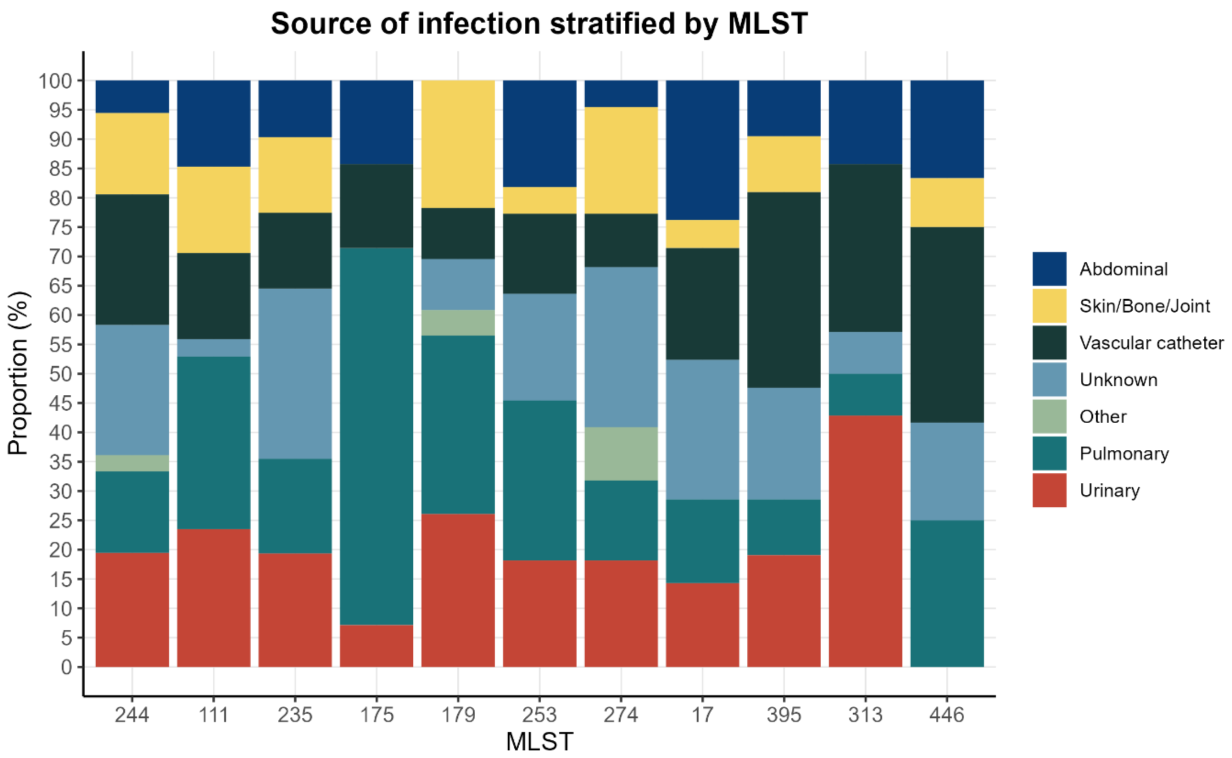

Supplementary Figure 4. Relationship between virulence clusters and sequence type

Circle plot illustrating the distribution of sequence types across clusters A-K for all 773 samples.

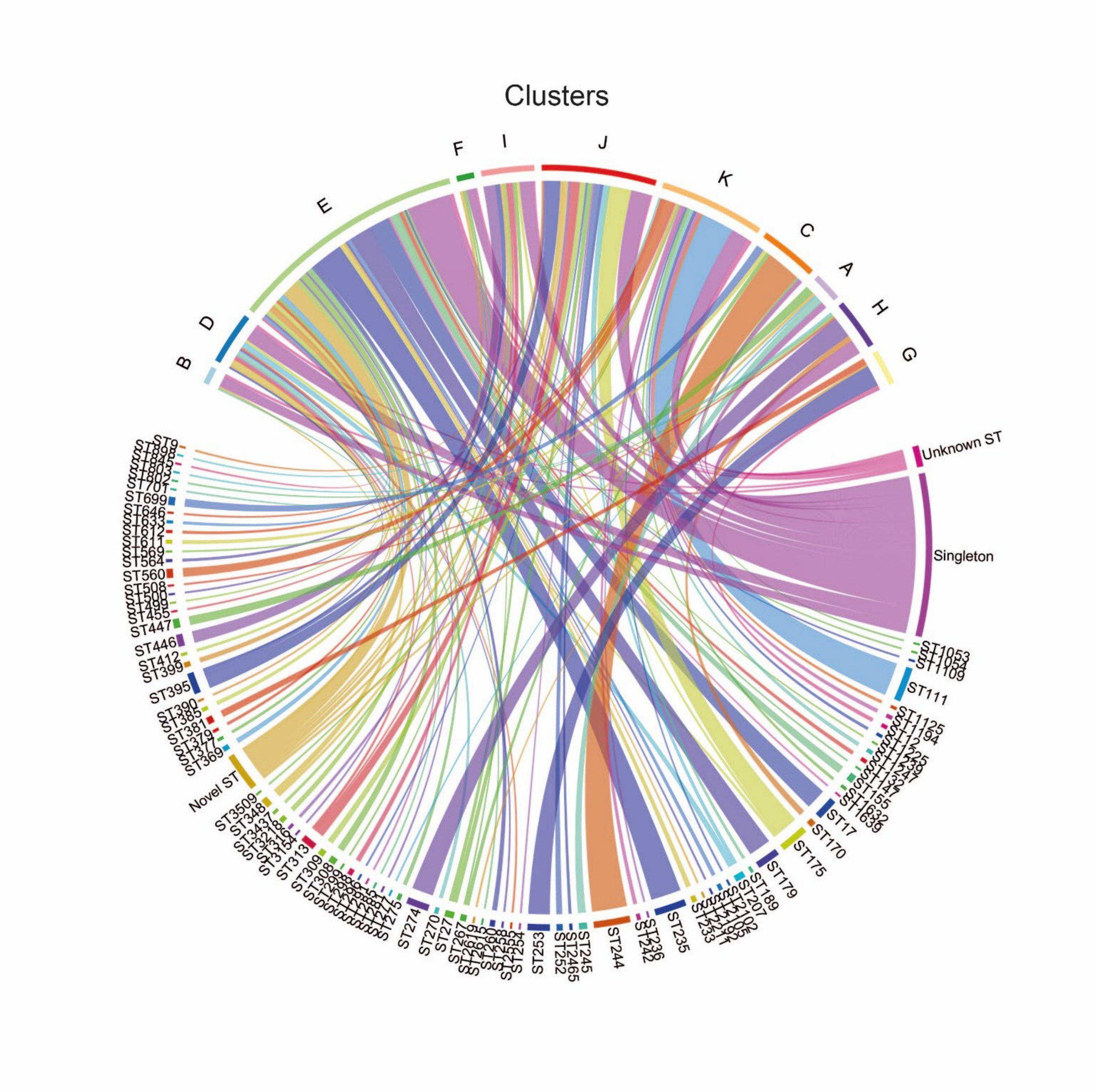

Supplementary Figure 5. Impact of genetic variation on the predictive machine learning models

Association between genomic virulence markers and patient outcomes, using varying gene identity thresholds to evaluate the impact of genetic variation. Panel a-b is based on genotyping with a gene identity threshold of 95%. As comparison, Panel b-c is based on genotyping with a gene identity threshold of 80% (same as shown in the main manuscript).

Panel a and c. Box plot (median [IQR]) of 4 random forest classifiers fitted to predict mortality or septic shock and assessed in the validation set. All models were based on different combinations of predictors. *Reference model:* patient characteristics alone (age, sex, comorbidity, immunosuppression, hospital department, and nosocomial infection), *Model 2:* combination of patient characteristics and all filtered and grouped virulence genes, *Model 3:* combination of patient characteristics and virulence genes selected by the Boruta algorithm, and *Model 4:* combination of patient characteristics and virulence or resistance genes selected by the Boruta algorithm. The plot shows the area under receiver operating characteristics (AUROC) for each of the 1000 random splits of the data into development and validation set, with a dashed line marking the median of the *Reference model*. **Panel b and d.** Box plot (median [IQR]) of the difference in AUROC between Model 2-4 and the Reference model for each of the 1000 splits, with a dashed line marking 0.00 difference. A positive difference occurs when the AUROC of the virulence model is greater than the Reference model. A negative difference occurs when the AUROC of the virulence model is less than the Reference model.

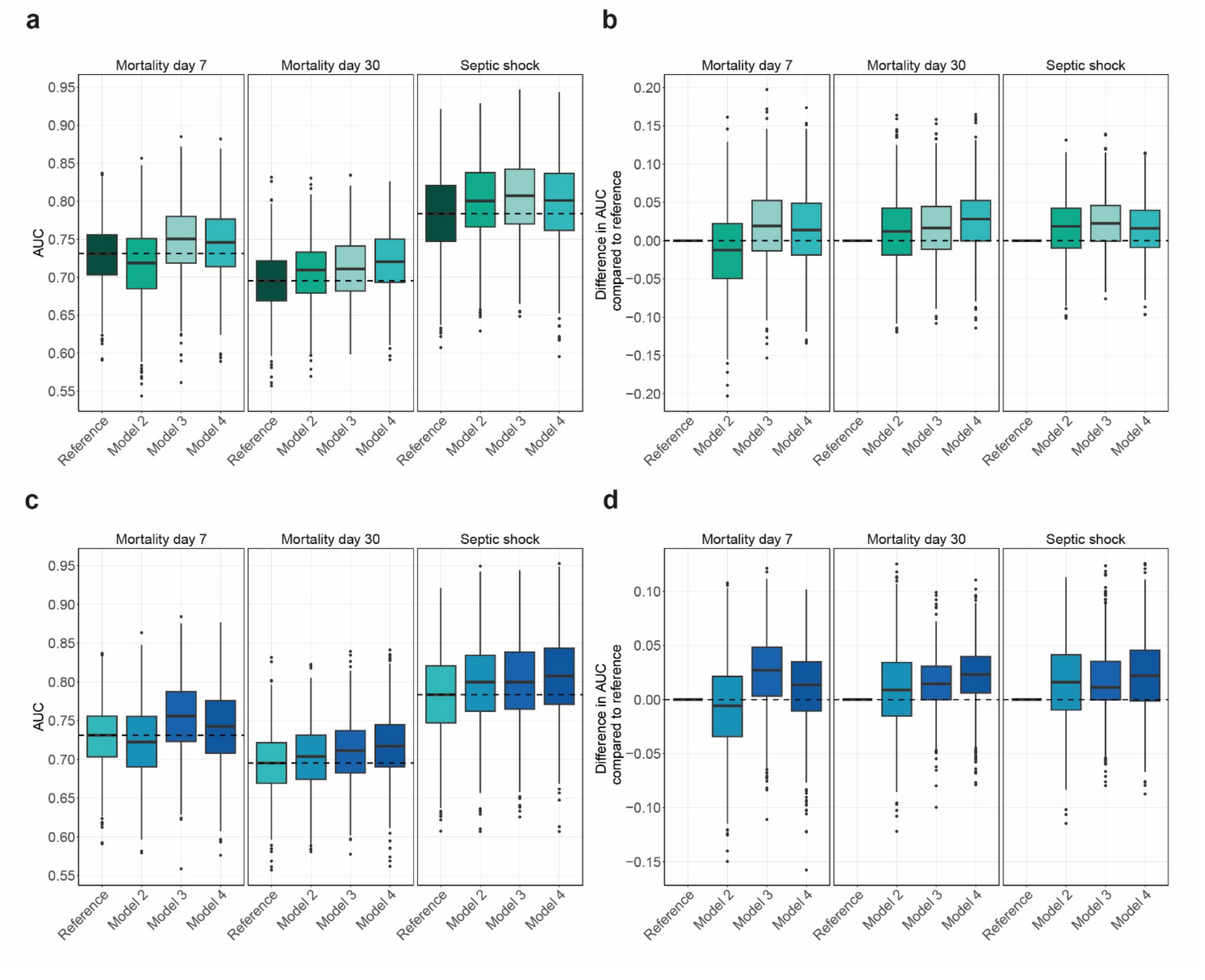

Supplementary Figure 6. Boruta feature selection output when including both virulence and resistance genes

The importance of specific virulence and resistance genes to predict mortality or septic shock based on the Boruta feature selection algorithm applied in the development set for each of the 1000 random splits of the data into development and validation set. Genes selected at least one time are shown on the y-axis. The heatmap color is based on the number of times a feature was selected ranging between 1-1000.

Abbreviations: Escherichia coli EFTu mutants conferring resistance to Pulvomycin (EF-Tu_PulR), Escherichia coli EFTu mutants conferring resistance to kirromycin (EF-Tu_KirR), Pseudomonas aeruginosa catB7 (catB7), and Pseudomonas aeruginosa oprD with mutation conferring resistance to imipenem (oprD_ImpR)

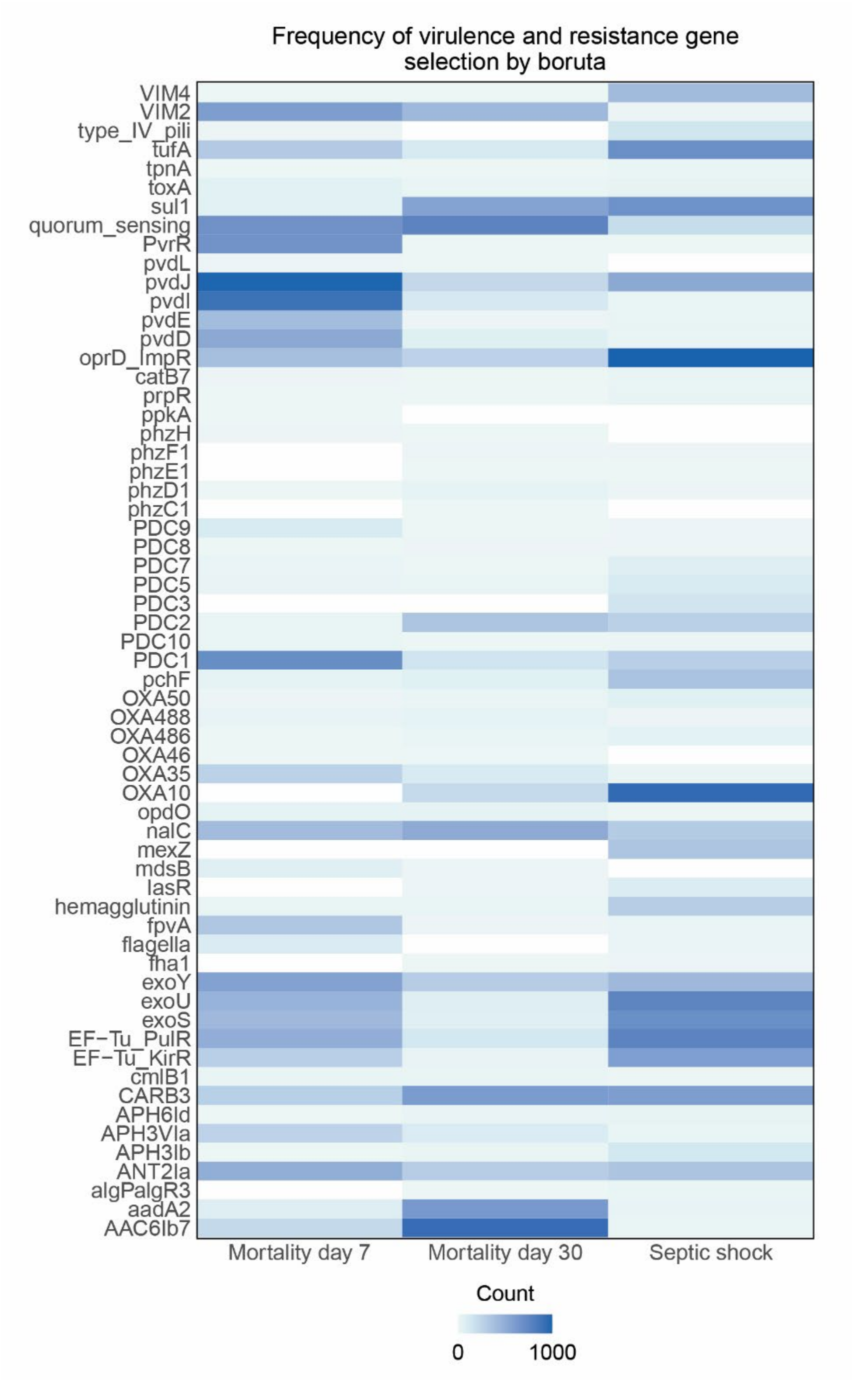

Supplementary Table 1. Time period when the study sites contributed with data

Study site	Study period (year)							
	2009	2010	2011	2012	2013	2014	2015	Total
Australia (n)								
Episodes	44	48	0	0	0	0	0	92
Episodes with strain*	40	46	0	0	0	0	0	86
Greece (n)								
Episodes	13	12	10	2	27	22	9	95
Episodes with strain*	7	12	9	2	24	21	9	84
Spain (Santander) (n)								
Episodes	19	22	37	24	33	26	23	184
Episodes with strain*	19	20	36	23	30	25	22	175
Spain (Seville) (n)								
Episodes	0	16	21	27	21	33	16	134
Episodes with strain*	0	16	21	27	21	33	16	134
Slovenia (n)								
Episodes	0	0	26	28	24	28	18	124
Episodes with strain*	0	0	26	27	24	28	18	123
Sweden (n)								
Episodes	0	30	39	29	39	41	29	207
Episodes with strain*	0	14	26	26	36	41	28	171

*Not all bacterial isolates survived either the storage at each institution or the transportation to Sweden where the whole genome sequence was performed.

Supplementary Table 2. Material and software resources

RESOURCE	SOURCE	IDENTIFIER
Bacterial and virus strains		
Pseudomonas aeruginosa	ATCC	ATCC 27853
Escherichia coli	ATCC	ATCC 25922
Chemicals, peptides, and recombinant proteins		
Mueller Hinton Agar	Thermo Fisher	Cat#R454086
Oxoid™ Meropenem Antimicrobial Susceptibility discs, 10μg	Thermo Fisher	Cat#CT0774B
Oxoid™ Imipenem Antimicrobial Susceptibility discs, 10μg	Thermo Fisher	Cat#CT0455B
Oxoid™ Ciprofloxacin Antimicrobial Susceptibility discs, 5μg	Thermo Fisher	Cat#CT0425B
Oxoid™ Amikacin Antimicrobial Susceptibility discs, 30μg	Thermo Fisher	Cat#CT0107B
Oxoid™ Tobramycin Antimicrobial Susceptibility discs, 10μg	Thermo Fisher	Cat#CT0056B
Oxoid™ Gentamicin Antimicrobial Susceptibility discs, 10μg	Thermo Fisher	Cat#CT0024B
Oxoid™ Piperacillin/Tazobactam Antimicrobial Susceptibility discs, 36μg	Thermo Fisher	Cat#CT1616B
Oxoid™ Ceftazidime Antimicrobial Susceptibility discs, 10μg	Thermo Fisher	Cat#CT1629B
Oxoid™ Aztreonam Antimicrobial Susceptibility discs, 30μg	Thermo Fisher	Cat#CT0264B
Commercial assays		
EZ1 DNA Tissue Kit	Qiagen	Cat#953034
Qubit dsDNA HS Assay Kit	Thermo Fisher	Cat#Q32851
Nextera XT DNA Library Prep Kit	Illumina	Cat#FC-131-1096
Deposited data		
Pseudomonas aeruginosa whole genome sequences	This paper	NCBI Sequence Read Archive: PRJNA1189087
Annotated virulence genome and resistance phenotype data	This paper	DOI: 10.17632/3h9gvbzz7x.2
Software and algorithms		
R Statistical Software (v4.3.1)	R Core Team	https://cran.r-project.org/src/base/R-4/
RStudio (v2023.06.2)	Posit Software	https://posit.co/download/rstudio-desktop/
microSALT (v2.8.12 to v 3.0.1)	SciLifeLab, Stockholm, Sweden	https://github.com/Clinical-Genomics/microSALT/
FastQC (v0.11.9)	Andrews S. (2010)[1]	https://github.com/s-andrews/FastQC
Trim Galore (v0.6.1)	Krueger F. (2015)[2]	https://github.com/FelixKrueger/TrimGalore
SPAdes (v3.14.1)	Bankevich A. et al. (2012)[3]	https://github.com/ablab/spades
PubMLST (accessed: March 2021)	PubMLST[4]	https://pubmlst.org/
BLAST+ (v2.9.0+)	NCBI[5]	https://ftp.ncbi.nlm.nih.gov/blast/executables/blast+/
MAFFT (v7.407)	Katoh K. et al. (2013)[6]	https://mafft.cbrc.jp/alignment/software/
FastTree (v2.1.10)	Price M. et al. (2009)[7]	http://www.microbesonline.org/fasttree/
Ggtree (v3.8.2)	Yu G. et al. (2017)[8]	https://bioconductor.org/packages/release/bioc/html/ggtree.html
Prodigal (v2.6.3)	Hyatt D. et al. (2010)[9]	https://github.com/hyattpd/Prodigal
Diamond (v2.0.4)	Buchfink, B. et al. (2021)[10]	https://github.com/bbuchfink/diamond
VFDB (accessed: March 2021)	Chen L. et al. (2005)[11]	http://www.mgc.ac.cn/VFs/
Victors (accessed: March 2021)	Sayers S. et al. (2019)[12]	https://phidias.us/victors/
Boruta (v8.0.0)	Kursa M. et al. (2010)[13]	https://cran.r-project.org/web/packages/Boruta/index.html
Caret (v6.0-94)	Kuhn M.	https://cran.r-project.org/web/packages/caret/index.html
Tidyverse (v2.0.0)	Wickham H.	https://cran.r-project.org/web/packages/tidyverse/index.html
Circlize (v0.4.15)	Gu Z. et al. (2014)[14]	https://cran.r-project.org/web/packages/circlize/index.html

Supplementary Table 3. Whole genome sequence quality control

Variable	Min	Max	Median	Percentile 25%	Percentile 75%
**Total Reads**	484653	15995342	6485564	5181732	8873282
Median Insert Size	183	519	332	274	382
Mapped rate (%)	33	97	87	82	91
Coverage 10X (%)	24	100	100	97	100

Supplementary Table 4. Relationship between bacterial genotype, multi-drug resistance and patient characteristics

Bacterial genotype	Number of isolates	Proportion of MDR[b]	Age (years), median (IQR)[c]	Proportion of chronic lung disease[d]	Proportion of vulnerable host[e]	Proportion of no underlying comorbidity[f]
Sequence type[a]						
ST244	36	0.00	68 (60-79)	0.25	0.50	0.08
ST111	34	0.85	62 (53-74)	0.15	0.41	0.26
ST235	31	0.65	71 (61-83)	0.03	0.65	0.16
ST175	28	0.71	60 (50-67)	0.39	0.64	0.07
ST179	23	0.04	65 (56-75)	0.09	0.52	0.22
ST253	22	0.09	68 (57-74)	0.27	0.41	0.23
ST274	22	0.00	73 (61-82)	0.23	0.41	0.05
ST395	21	0.05	71 (52-78)	0.19	0.48	0.14
ST17	21	0.29	63 (53-75)	0.14	0.38	0.29
ST313	14	0.21	71 (68-84)	0.00	0.43	0.14
ST446	12	0.08	55 (47-58)	0.25	0.58	0.25
Virulence cluster						
A	29	0,07	62 (51.70	0,10	0,52	0,24
B	18	0,06	70 (66-78)	0,50	0,56	0,06
C	59	0,03	69 (58-80)	0,25	0,47	0,10
D	53	0,13	71 (58-80)	0,23	0,45	0,17
E	237	0,16	67 (57-78)	0,14	0,45	0,19
F	18	0,00	77 (65-83)	0,11	0,33	0,22
G	36	0,06	69 (58-74)	0,28	0,44	0,22
H	51	0,00	67 (57-80)	0,10	0,45	0,14
I	53	0,09	66 (55-74)	0,19	0,43	0,17
J	114	0,21	66 (53-77)	0,18	0,52	0,15
K	105	0,29	69 (58-76)	0,17	0,50	0,18
T3SS						
exoU+	162	0.22	68 (57-77)	0.15	0.52	0.16
exoU-	611	0.13	68 (57-78)	0.19	0.46	0.17
exoS+	598	0.13	68 (57-78)	0.18	0.45	0.17
exoS-	175	0.20	68 (57-77)	0.17	0.54	0.15
exoY+	666	0.13	68 (56-58)	0.19	0.46	0.18
exoY-	107	0.26	68 (60-80)	0.12	0.53	0.13

[a]Only clones with >10 isolates are show.

[b]MDR defined as phenotypic resistance to 3 or more antimicrobial drugs from different drug classes. Sequence type, p-value=0.0001; virulence cluster, p-value=0.0001; exoU+/-, p=0.006; exoS+/-, p=0.03; and exoY+/-, p=0.0004.

[c]Sequence type, p-value=0.009; and virulence cluster, p-value=0.15.

[d]Sequence type, p-value=0.01; virulence cluster, p-value = 0.01; exoU+/-, p=0.43; exoS+/-, p=0.69; and exoY+/-, p=0.12.

[e]Sequence type, p-value=0.50; virulence cluster, p-value=0.93; exoU+/-, p=0.15; exoS+/-, p=0.04; and exoY+/-, p=0.20.

[f]Sequence type, p-value=0.24; virulence cluster, p-value=0.72; exoU+/-, p=0.82; exoS+/-, p=0.62; and exoY+/-, p=0.31.

Abbreviations: Multidrug-resistance (MDR), Inter Quartile Range (IQR), Confidence Interval (CI) and Type III Secretion System (T3SS).

Supplementary Table 5. Relationship between virulence clusters and sequence type in *P. aeruginosa*

	Clusters											
	Overall	A	B	C	D	E	F	G	H	I	J	K
Count	373	17	0	48	20	90	2	29	28	27	69	43
ST, n (%)												
ST27	9 (2)	0 (0)	0 (0)	0 (0)	0 (0)	0 (0)	0 (0)	0 (0)	0 (0)	4 (15)	5 (7)	0 (0)
ST348	9 (2)	0 (0)	0 (0)	0 (0)	6 (30)	0 (0)	0 (0)	0 (0)	0 (0)	0 (0)	3 (4)	0 (0)
ST447	9 (2)	9 (53)	0 (0)	0 (0)	0 (0)	0 (0)	0 (0)	0 (0)	0 (0)	0 (0)	0 (0)	0 (0)
ST560	9 (2)	0 (0)	0 (0)	0 (0)	0 (0)	0 (0)	0 (0)	0 (0)	0 (0)	0 (0)	0 (0)	9 (21)
ST155	8 (2)	0 (0)	0 (0)	0 (0)	0 (0)	8 (9)	0 (0)	0 (0)	0 (0)	0 (0)	0 (0)	0 (0)
ST245	8 (2)	8 (47)	0 (0)	0 (0)	0 (0)	0 (0)	0 (0)	0 (0)	0 (0)	0 (0)	0 (0)	0 (0)
ST699	8 (2)	0 (0)	0 (0)	8 (17)	0 (0)	0 (0)	0 (0)	0 (0)	0 (0)	0 (0)	0 (0)	0 (0)
ST308	7 (2)	0 (0)	0 (0)	0 (0)	0 (0)	7 (8)	0 (0)	0 (0)	0 (0)	0 (0)	0 (0)	0 (0)
ST309	7 (2)	0 (0)	0 (0)	0 (0)	0 (0)	0 (0)	0 (0)	0 (0)	0 (0)	1 (4)	6 (9)	0 (0)
ST381	7 (2)	0 (0)	0 (0)	0 (0)	0 (0)	0 (0)	0 (0)	7 (24)	0 (0)	0 (0)	0 (0)	0 (0)
ST170	6 (2)	0 (0)	0 (0)	0 (0)	0 (0)	0 (0)	0 (0)	0 (0)	6 (21)	0 (0)	0 (0)	0 (0)
ST252	6 (2)	0 (0)	0 (0)	0 (0)	0 (0)	0 (0)	0 (0)	0 (0)	0 (0)	0 (0)	6 (9)	0 (0)
ST267	6 (2)	0 (0)	0 (0)	4 (8)	0 (0)	0 (0)	2 (100)	0 (0)	0 (0)	0 (0)	0 (0)	0 (0)
ST244	36 (10)	0 (0)	0 (0)	36 (75)	0 (0)	0 (0)	0 (0)	0 (0)	0 (0)	0 (0)	0 (0)	0 (0)
ST111	34 (9)	0 (0)	0 (0)	0 (0)	0 (0)	0 (0)	0 (0)	0 (0)	0 (0)	0 (0)	0 (0)	34 (79)
ST235	31 (8)	0 (0)	0 (0)	0 (0)	0 (0)	31 (34)	0 (0)	0 (0)	0 (0)	0 (0)	0 (0)	0 (0)
ST175	28 (8)	0 (0)	0 (0)	0 (0)	3 (15)	0 (0)	0 (0)	0 (0)	0 (0)	3 (11)	22 (32)	0 (0)
ST179	23 (6)	0 (0)	0 (0)	0 (0)	0 (0)	23 (26)	0 (0)	0 (0)	0 (0)	0 (0)	0 (0)	0 (0)
ST253	22 (6)	0 (0)	0 (0)	0 (0)	0 (0)	0 (0)	0 (0)	22 (76)	0 (0)	0 (0)	0 (0)	0 (0)
ST274	22 (6)	0 (0)	0 (0)	0 (0)	0 (0)	0 (0)	0 (0)	0 (0)	22 (79)	0 (0)	0 (0)	0 (0)
ST17	21 (6)	0 (0)	0 (0)	0 (0)	0 (0)	21 (23)	0 (0)	0 (0)	0 (0)	0 (0)	0 (0)	0 (0)
ST395	21 (6)	0 (0)	0 (0)	0 (0)	2 (10)	0 (0)	0 (0)	0 (0)	0 (0)	5 (19)	14 (20)	0 (0)
ST313	14 (4)	0 (0)	0 (0)	0 (0)	3 (15)	0 (0)	0 (0)	0 (0)	0 (0)	2 (7)	9 (13)	0 (0)
ST446	12 (3)	0 (0)	0 (0)	0 (0)	0 (0)	0 (0)	0 (0)	0 (0)	0 (0)	12 (44)	0 (0)	0 (0)
ST207	10 (3)	0 (0)	0 (0)	0 (0)	6 (30)	0 (0)	0 (0)	0 (0)	0 (0)	0 (0)	4 (6)	0 (0)

Abbreviations: Sequence Type (ST), Numbers (n).

Supplementary Table 6. Univariable logistic regression: 7-day mortality

Exposure	Total obs.	Total exposure	Events per exposure	Proportion of events per exposure	Odds ratio	CI 2.5%	CI 97.5%	P-value
clusterA	773	29	1	0.03	0.19	0.03	1.39	0.10
clusterB	773	18	2	0.11	0.67	0.15	2.97	0.60
clusterC	773	59	7	0.12	0.72	0.32	1.62	0.42
clusterD	773	53	11	0.21	1.47	0.73	2.94	0.28
clusterE	773	237	43	0.18	1.32	0.88	1.99	0.18
clusterF	773	18	2	0.11	0.67	0.15	2.97	0.60
clusterG	773	36	4	0.11	0.67	0.23	1.93	0.46
clusterH	773	51	3	0.06	0.32	0.10	1.06	0.06
clusterI	773	53	15	0.28	2.31	1.23	4.35	***0.01***
clusterJ	773	114	14	0.12	0.73	0.40	1.33	0.30
clusterK	773	105	18	0.17	1.15	0.66	1.99	0.62
ST244	773	36	5	0.14	0.87	0.33	2.29	0.78
ST111	773	34	10	0.29	2.38	1.11	5.12	***0.03***
ST235	773	31	10	0.32	2.74	1.25	5.97	***0.01***
ST175	773	28	11	0.39	3.78	1.72	8.28	***0.00***
ST179	773	23	3	0.13	0.81	0.24	2.77	0.74
ST253	773	22	1	0.05	0.25	0.03	1.90	0.18
ST274	773	22	2	0.09	0.54	0.12	2.32	0.41
ST17	773	21	2	0.10	0.57	0.13	2.46	0.45
ST395	773	21	5	0.24	1.73	0.62	4.82	0.29
ST313	773	14	1	0.07	0.41	0.05	3.19	0.40
ST446	773	12	3	0.25	1.83	0.49	6.88	0.37
algPalgR3	773	749	116	0.15	0.92	0.31	2.73	0.88
exoS	773	598	91	0.15	0.90	0.57	1.43	0.66
exoU	773	162	26	0.16	1.05	0.65	1.69	0.84
exoY	773	666	96	0.14	0.58	0.35	0.96	***0.04***
fha1	773	673	108	0.16	1.40	0.74	2.65	0.30
ppkA	773	754	117	0.16	0.98	0.28	3.41	0.97
pchF	773	745	117	0.16	1.55	0.46	5.23	0.48
fpvA	773	334	53	0.16	1.05	0.71	1.55	0.82
pvdD	773	226	35	0.15	1.00	0.65	1.53	0.99
pvdE	773	332	53	0.16	1.06	0.72	1.57	0.77
pvdI	773	254	39	0.15	0.98	0.65	1.49	0.93
pvdJ	773	217	30	0.14	0.83	0.53	1.30	0.42
pvdL	773	754	117	0.16	0.98	0.28	3.41	0.97
phzC1	773	474	64	0.14	0.68	0.46	1.00	0.05
phzD1	773	596	88	0.15	0.78	0.50	1.22	0.29
phzE1	773	348	52	0.15	0.92	0.62	1.37	0.69
phzF1	773	625	97	0.16	1.00	0.61	1.64	1.00
phzG1	773	695	107	0.15	0.91	0.48	1.71	0.77
phzH	773	752	118	0.16	1.77	0.41	7.69	0.45
toxA	773	756	115	0.15	0.43	0.15	1.25	0.12
tufA	773	622	100	0.16	1.25	0.75	2.10	0.39
flagella	773	293	35	0.12	0.63	0.41	0.96	***0.03***
type_IV_pili	773	663	111	0.17	2.26	1.11	4.60	***0.02***
rhamnolipid	773	745	110	0.15	0.31	0.14	0.69	***0.00***
lasR	773	722	115	0.16	1.74	0.68	4.48	0.25
hemagglutinin	773	344	45	0.13	0.71	0.48	1.06	0.09
prpR	773	541	86	0.16	1.10	0.72	1.69	0.66
opdO	773	738	115	0.16	1.11	0.42	2.91	0.84
tpnA	773	281	47	0.17	1.15	0.77	1.72	0.49

Abbreviations: Observations (obs), Sequence Type (ST), Confidence Interval (CI)

Supplementary Table 7. Univariable logistic regression: 30-day mortality

Exposure	Total obs.	Total exposure	Events per exposure	Proportion of events per exposure	Odds ratio	CI 2.5%	CI 97.5%	P-value
clusterA	773	29	7	0.24	1.03	0.43	2.46	0.94
clusterB	773	18	4	0.22	0.93	0.30	2.85	0.89
clusterC	773	59	11	0.19	0.73	0.37	1.43	0.36
clusterD	773	53	14	0.26	1.18	0.63	2.22	0.61
clusterE	773	237	60	0.25	1.15	0.81	1.64	0.44
clusterF	773	18	2	0.11	0.40	0.09	1.75	0.22
clusterG	773	36	7	0.19	0.78	0.33	1.80	0.55
clusterH	773	51	8	0.16	0.59	0.27	1.27	0.18
clusterI	773	53	18	0.34	1.74	0.96	3.16	0.07
clusterJ	773	114	18	0.16	0.57	0.33	0.96	***0.04***
clusterK	773	105	33	0.31	1.60	1.02	2.51	***0.04***
ST244	773	36	7	0.19	0.78	0.33	1.80	0.55
ST111	773	34	13	0.38	2.09	1.02	4.26	***0.04***
ST235	773	31	15	0.48	3.23	1.56	6.67	***0.00***
ST175	773	28	14	0.50	3.43	1.61	7.35	***0.00***
ST179	773	23	6	0.26	1.15	0.45	2.96	0.77
ST253	773	22	4	0.18	0.72	0.24	2.14	0.55
ST274	773	22	4	0.18	0.72	0.24	2.14	0.55
ST17	773	21	4	0.19	0.76	0.25	2.28	0.62
ST395	773	21	5	0.24	1.02	0.37	2.81	0.98
ST313	773	14	1	0.07	0.25	0.03	1.89	0.18
ST446	773	12	4	0.33	1.64	0.49	5.50	0.43
algPalgR3	773	749	177	0.24	1.18	0.43	3.19	0.75
exoS	773	598	137	0.23	0.86	0.58	1.27	0.44
exoU	773	162	41	0.25	1.13	0.76	1.69	0.55
exoY	773	666	147	0.22	0.58	0.37	0.91	**0.02**
fha1	773	673	165	0.25	1.59	0.91	2.75	0.10
ppkA	773	754	178	0.24	1.16	0.38	3.54	0.80
pchF	773	745	177	0.24	1.43	0.54	3.83	0.47
fpvA	773	334	70	0.21	0.77	0.55	1.09	0.14
pvdD	773	226	49	0.22	0.86	0.59	1.25	0.43
pvdE	773	332	70	0.21	0.78	0.56	1.10	0.16
pvdI	773	254	53	0.21	0.80	0.55	1.15	0.22
pvdJ	773	217	40	0.18	0.66	0.44	0.98	***0.04***
pvdL	773	754	178	0.24	1.16	0.38	3.54	0.80
phzC1	773	474	99	0.21	0.69	0.49	0.96	***0.03***
phzD1	773	596	130	0.22	0.67	0.46	0.98	***0.04***
phzE1	773	348	82	0.24	1.00	0.72	1.40	0.99
phzF1	773	625	150	0.24	1.14	0.74	1.76	0.54
phzG1	773	695	163	0.23	0.95	0.55	1.64	0.86
phzH	773	752	179	0.24	1.87	0.55	6.44	0.32
toxA	773	756	176	0.23	0.56	0.20	1.53	0.25
tufA	773	622	153	0.25	1.37	0.88	2.14	0.16
flagella	773	293	68	0.23	0.97	0.69	1.37	0.86
type_IV_pili	773	663	163	0.25	1.56	0.92	2.64	0.10
rhamnolipid	773	745	168	0.23	0.29	0.14	0.62	***0.00***
lasR	773	722	172	0.24	1.28	0.63	2.61	0.49
hemagglutinin	773	344	69	0.20	0.70	0.50	0.99	***0.04***
prpR	773	541	131	0.24	1.13	0.79	1.64	0.50
opdO	773	738	173	0.23	0.88	0.41	1.92	0.76
tpnA	773	281	72	0.26	1.20	0.85	1.68	0.30

Abbreviations: Observations (obs), Sequence Type (ST), Confidence Interval (CI)

Supplementary Table 8. Univariable logistic regression: septic shock

Exposure	Total obs.	Total exposure	Events per exposure	Proportion of events per exposure	Odds ratio	CI 2.5%	CI 97.5%	P-value
clusterA	639	27	5	0.19	1.04	0.38	2.80	0.94
clusterB	639	15	1	0.07	0.32	0.04	2.45	0.27
clusterC	639	44	6	0.14	0.70	0.29	1.71	0.44
clusterD	639	46	7	0.15	0.81	0.35	1.85	0.61
clusterE	639	192	39	0.20	1.24	0.81	1.91	0.32
clusterF	639	15	1	0.07	0.32	0.04	2.45	0.27
clusterG	639	29	7	0.24	1.48	0.62	3.55	0.38
clusterH	639	37	6	0.16	0.88	0.36	2.15	0.77
clusterI	639	39	4	0.10	0.50	0.18	1.45	0.20
clusterJ	639	102	20	0.20	1.13	0.66	1.94	0.64
clusterK	639	93	19	0.20	1.20	0.69	2.09	0.51
ST244	639	30	1	0.03	0.15	0.02	1.11	0.06
ST111	639	33	10	0.30	2.07	0.96	4.49	0.06
ST235	639	30	9	0.30	2.03	0.91	4.56	0.09
ST175	639	28	10	0.36	2.68	1.20	5.96	***0.02***
ST179	639	17	4	0.24	1.42	0.45	4.43	0.55
ST253	639	19	3	0.16	0.85	0.24	2.97	0.80
ST274	639	15	2	0.13	0.70	0.15	3.13	0.64
ST17	639	13	1	0.08	0.37	0.05	2.90	0.35
ST395	639	21	2	0.10	0.47	0.11	2.05	0.32
ST313	639	12	2	0.17	0.91	0.20	4.21	0.90
ST446	639	10	1	0.10	0.50	0.06	4.00	0.52
algPalgR3	639	618	114	0.18	4.52	0.60	34.05	0.14
exoS	639	490	83	0.17	0.75	0.47	1.18	0.21
exoU	639	138	32	0.23	1.52	0.96	2.41	0.07
exoY	639	545	92	0.17	0.63	0.37	1.06	0.08
fha1	639	563	98	0.17	0.73	0.41	1.31	0.29
ppkA	639	622	114	0.18	3.59	0.47	27.35	0.22
pchF	639	617	115	0.19	>100	0.00	Inf	0.98
fpvA	639	276	45	0.16	0.82	0.54	1.23	0.33
pvdD	639	190	36	0.19	1.09	0.71	1.69	0.68
pvdE	639	274	45	0.16	0.83	0.55	1.25	0.37
pvdI	639	215	37	0.17	0.92	0.60	1.42	0.71
pvdJ	639	172	23	0.13	0.63	0.38	1.03	0.07
pvdL	639	624	115	0.18	>100	0.00	Inf	0.98
phzC1	639	384	63	0.16	0.77	0.51	1.15	0.20
phzD1	639	492	89	0.18	1.03	0.63	1.66	0.91
phzE1	639	294	59	0.20	1.30	0.86	1.94	0.21
phzF1	639	524	93	0.18	0.91	0.54	1.53	0.73
phzG1	639	573	105	0.18	1.26	0.62	2.54	0.53
phzH	639	622	112	0.18	1.02	0.29	3.63	0.97
toxA	639	628	115	0.18	>100	0.00	Inf	0.98
tufA	639	523	102	0.20	1.92	1.04	3.55	***0.04***
flagella	639	242	43	0.18	0.98	0.64	1.48	0.91
type_IV_pili	639	555	100	0.18	1.01	0.56	1.84	0.97
rhamnolipid	639	612	107	0.17	0.50	0.21	1.18	0.11
lasR	639	597	105	0.18	0.68	0.33	1.43	0.31
hemagglutinin	639	280	38	0.14	0.58	0.38	0.88	***0.01***
prpR	639	460	77	0.17	0.75	0.48	1.15	0.19
opdO	639	611	111	0.18	1.33	0.45	3.92	0.60
tpnA	639	241	50	0.21	1.34	0.89	2.02	0.16

Abbreviations: Observations (obs), Sequence Type (ST), Confidence Interval (CI)

Supplementary Table 9. Multivariable logistic regression: 7-day mortality

Exposure	Total obs.	Odds ratio	CI 2.5%	CI 97.5%	P-value
clusterA	772	0.08	0.01	0.75	***0.03***
clusterB	772	0.81	0.16	4.02	0.79
clusterC	772	0.67	0.26	1.70	0.40
clusterD	772	1.03	0.44	2.44	0.94
clusterF	772	0.51	0.09	2.97	0.46
clusterG	772	0.41	0.12	1.40	0.15
clusterH	772	0.32	0.09	1.18	0.09
clusterI	772	2.20	0.98	4.94	0.06
clusterJ	772	0.44	0.20	0.94	***0.03***
clusterK	772	0.66	0.33	1.33	0.25
ST244	772	1.54	0.54	4.34	0.42
ST111	772	1.49	0.60	3.72	0.39
ST235	772	3.12	1.15	8.45	***0.03***
ST175	772	3.38	1.21	9.43	***0.02***
ST179	772	1.32	0.33	5.20	0.69
ST253	772	0.28	0.03	2.32	0.24
ST274	772	0.75	0.15	3.73	0.72
ST17	772	1.36	0.29	6.42	0.70
ST395	772	2.30	0.64	8.28	0.20
ST313	772	1.06	0.12	9.55	0.96
ST446	772	4.82	0.92	25.14	0.06
exoS	772	0.86	0.51	1.45	0.56
exoU	772	1.00	0.58	1.73	0.99
exoY	772	0.62	0.34	1.11	0.11
pvdJ	772	0.90	0.55	1.48	0.67
phzC1	772	0.86	0.55	1.35	0.52
phzD1	772	0.86	0.52	1.43	0.56
tufA	772	1.32	0.74	2.33	0.35
flagella	772	0.56	0.35	0.90	***0.02***
type_IV_pili	772	2.06	0.95	4.44	0.07
rhamnolipid	772	0.36	0.13	0.98	***0.05***
hemagglutinin	772	0.93	0.59	1.46	0.75

Logistic regression model with adjustments for geographical site, age group, sex, charlson comorbidity index group, immunosuppressed state, department of hospitalization and nosocomial infection. The final cohort consisted of n=772 episodes due to missing data on department of hospitalization for one episode. ST was modelled as a categorical variable with all other STs used as reference. Virulence cluster was modelled as a categorical variable with the largest cluster, Cluster E (n=237), used as reference.

Abbreviations: Observations (obs), Sequence Type (ST), Confidence Interval (CI)

Supplementary Table 10. Multivariable logistic regression: 30-day mortality

Exposure	Total obs.	Odds ratio	CI 2.5%	CI 97.5%	P-value
clusterA	772	0.76	0.27	2.15	0.61
clusterB	772	1.06	0.31	3.60	0.93
clusterC	772	0.76	0.35	1.67	0.49
clusterD	772	0.97	0.45	2.09	0.93
clusterF	772	0.28	0.05	1.56	0.15
clusterG	772	0.57	0.21	1.55	0.27
clusterH	772	0.62	0.25	1.52	0.30
clusterI	772	1.89	0.89	4.00	0.10
clusterJ	772	0.40	0.20	0.79	***0.01***
clusterK	772	1.06	0.60	1.89	0.84
ST244	772	1.36	0.54	3.37	0.51
ST111	772	1.23	0.54	2.84	0.62
ST235	772	3.51	1.42	8.68	***0.01***
ST175	772	3.16	1.21	8.26	***0.02***
ST179	772	1.93	0.66	5.58	0.23
ST253	772	0.80	0.23	2.74	0.72
ST274	772	0.87	0.26	2.98	0.83
ST17	772	1.43	0.43	4.73	0.56
ST395	772	1.12	0.31	4.05	0.86
ST313	772	0.48	0.06	3.98	0.49
ST446	772	3.69	0.86	15.81	0.08
exoS	772	0.82	0.52	1.28	0.38
exoU	772	1.09	0.69	1.73	0.71
exoY	772	0.62	0.37	1.04	0.07
pvdJ	772	0.69	0.45	1.07	0.10
phzC1	772	0.82	0.56	1.20	0.31
phzD1	772	0.75	0.49	1.15	0.19
tufA	772	1.49	0.90	2.45	0.12
flagella	772	0.92	0.62	1.34	0.65
type_IV_pili	772	1.41	0.79	2.51	0.25
rhamnolipid	772	0.33	0.13	0.83	***0.02***
hemagglutinin	772	0.88	0.60	1.30	0.52

Logistic regression model with adjustments for geographical site, age group, sex, charlson comorbidity index group, immunosuppressed state, department of hospitalization and nosocomial infection. The final cohort consisted of n=772 episodes due to missing data on department of hospitalization for one episode. ST was modelled as a categorical variable with all other STs used as reference. Virulence cluster was modelled as a categorical variable with the largest cluster, Cluster E (n=237), used as reference.

Abbreviations: Observations (obs), Sequence Type (ST), Confidence Interval (CI)

Supplementary Table 11. Multivariable logistic regression: septic shock

Exposure	Total obs.	Odds ratio	CI 2.5%	CI 97.5%	P-value
clusterA	638	0.74	0.19	2.82	0.66
clusterB	638	0.68	0.08	5.63	0.72
clusterC	638	0.82	0.26	2.57	0.73
clusterD	638	0.58	0.20	1.72	0.33
clusterF	638	0.27	0.03	2.89	0.28
clusterG	638	0.86	0.25	2.91	0.81
clusterH	638	1.06	0.33	3.41	0.93
clusterI	638	0.38	0.11	1.40	0.15
clusterJ	638	0.63	0.29	1.38	0.25
clusterK	638	0.68	0.31	1.49	0.33
ST244	638	0.46	0.06	3.59	0.46
ST111	638	0.62	0.23	1.65	0.33
ST235	638	2.86	0.93	8.80	0.07
ST175	638	1.00	0.33	3.02	1.00
ST179	638	1.97	0.45	8.73	0.37
ST253	638	0.72	0.14	3.67	0.70
ST274	638	1.72	0.28	10.77	0.56
ST17	638	1.13	0.14	9.39	0.91
ST395	638	0.50	0.08	3.01	0.45
ST313	638	2.28	0.38	13.68	0.37
ST446	638	0.65	0.06	7.22	0.72
exoS	638	0.52	0.29	0.95	***0.03***
exoU	638	1.99	1.08	3.64	***0.03***
exoY	638	0.41	0.21	0.81	***0.01***
pvdJ	638	0.59	0.32	1.08	0.09
phzC1	638	1.01	0.60	1.70	0.97
phzD1	638	1.04	0.56	1.92	0.91
tufA	638	2.96	1.38	6.34	***0.01***
flagella	638	0.95	0.57	1.60	0.86
type_IV_pili	638	0.78	0.36	1.66	0.52
rhamnolipid	638	1.24	0.40	3.83	0.71
hemagglutinin	638	0.82	0.48	1.39	0.45

Logistic regression model with adjustments for geographical site, age group, sex, charlson comorbidity index group, immunosuppressed state, department of hospitalization and nosocomial infection. The final cohort consisted of n=638 episodes due to missing data on septic shock from one study site (Seville). ST was modelled as a categorical variable with all other STs used as reference. Virulence cluster was modelled as a categorical variable with the largest cluster, Cluster E (n=192), used as reference.

Abbreviations: Observations (obs), Sequence Type (ST), Confidence Interval (CI)

Supplementary Table 12. Multivariable logistic regression adjusted for appropriate empiric antibiotic treatment: 7-day mortality

Exposure	Total obs.	Odds ratio	CI 2.5%	CI 97.5%	P-value
clusterA	772	0.07	0.01	0.67	***0.02***
clusterB	772	0.78	0.16	3.89	0.77
clusterC	772	0.68	0.26	1.76	0.43
clusterD	772	1.01	0.42	2.40	0.98
clusterF	772	0.56	0.10	3.21	0.51
clusterG	772	0.43	0.12	1.48	0.18
clusterH	772	0.32	0.09	1.18	0.09
clusterI	772	2.26	1.00	5.11	***0.05***
clusterJ	772	0.44	0.21	0.95	***0.04***
clusterK	772	0.67	0.33	1.35	0.26
ST244	772	1.57	0.55	4.52	0.40
ST111	772	1.53	0.61	3.81	0.37
ST235	772	3.18	1.17	8.66	***0.02***
ST175	772	3.36	1.20	9.45	***0.02***
ST179	772	1.20	0.31	4.63	0.79
ST253	772	0.30	0.04	2.51	0.27
ST274	772	0.75	0.15	3.69	0.72
ST17	772	1.42	0.30	6.76	0.66
ST395	772	2.41	0.68	8.59	0.18
ST313	772	0.99	0.11	8.90	0.99
ST446	772	5.29	1.02	27.47	***0.05***

Logistic regression model with adjustments for geographical site, age group, sex, charlson comorbidity index group, immunosuppressed state, department of hospitalization, nosocomial infection, and appropriate empiric antibiotic treatment. The final cohort consisted of n=772 episodes due to missing data on department of hospitalization for one episode. ST was modelled as a categorical variable with all other STs used as reference. Virulence cluster was modelled as a categorical variable with the largest cluster, Cluster E (n=237), used as reference.

Abbreviations: Observations (obs), Sequence Type (ST), Confidence Interval (CI)

Supplementary Table 13. Multivariable logistic regression adjusted for appropriate empiric antibiotic treatment: 30-day mortality

Exposure	Total obs.	Odds ratio	CI 2.5%	CI 97.5%	P-value
clusterA	772	0.75	0.27	2.13	0.59
clusterB	772	1.04	0.31	3.55	0.95
clusterC	772	0.77	0.35	1.69	0.51
clusterD	772	0.95	0.44	2.07	0.91
clusterF	772	0.29	0.05	1.59	0.15
clusterG	772	0.58	0.21	1.58	0.29
clusterH	772	0.62	0.25	1.52	0.30
clusterI	772	1.90	0.90	4.02	0.09
clusterJ	772	0.40	0.20	0.79	***0.01***
clusterK	772	1.07	0.60	1.9	0.82
ST244	772	1.37	0.55	3.43	0.50
ST111	772	1.24	0.54	2.86	0.61
ST235	772	3.54	1.43	8.77	***0.01***
ST175	772	3.16	1.21	8.25	***0.02***
ST179	772	1.85	0.64	5.35	0.25
ST253	772	0.82	0.24	2.81	0.75
ST274	772	0.87	0.26	2.95	0.82
ST17	772	1.45	0.43	4.81	0.55
ST395	772	1.13	0.31	4.08	0.85
ST313	772	0.47	0.06	3.88	0.48
ST446	772	3.78	0.88	16.18	0.07

Logistic regression model with adjustments for geographical site, age group, sex, charlson comorbidity index group, immunosuppressed state, department of hospitalization, nosocomial infection, and appropriate empiric antibiotic treatment. The final cohort consisted of n=772 episodes due to missing data on department of hospitalization for one episode. ST was modelled as a categorical variable with all other STs used as reference. Virulence cluster was modelled as a categorical variable with the largest cluster, Cluster E (n=237), used as reference.

Abbreviations: Observations (obs), Sequence Type (ST), Confidence Interval (CI)

Supplementary Table 14. Multivariable logistic regression adjusted for appropriate empiric antibiotic treatment: septic shock

Exposure	Total obs.	Odds ratio	CI 2.5%	CI 97.5%	P-value
clusterA	638	0.76	0.20	2.87	0.68
clusterB	638	0.69	0.08	5.70	0.73
clusterC	638	0.82	0.26	2.57	0.74
clusterD	638	0.61	0.20	1.81	0.37
clusterF	638	0.26	0.02	2.82	0.27
clusterG	638	0.83	0.25	2.82	0.77
clusterH	638	1.07	0.33	3.49	0.92
clusterI	638	0.38	0.10	1.37	0.14
clusterJ	638	0.63	0.29	1.37	0.24
clusterK	638	0.67	0.31	1.48	0.32
ST244	638	0.45	0.06	3.54	0.45
ST111	638	0.61	0.23	1.62	0.32
ST235	638	2.87	0.93	8.89	0.07
ST175	638	1.02	0.34	3.06	0.97
ST179	638	2.10	0.46	9.60	0.34
ST253	638	0.70	0.14	3.56	0.67
ST274	638	1.79	0.29	11.22	0.53
ST17	638	1.12	0.13	9.38	0.92
ST395	638	0.50	0.08	3.01	0.45
ST313	638	2.35	0.39	14.29	0.35
ST446	638	0.64	0.06	7.13	0.71

Logistic regression model with adjustments for geographical site, age group, sex, charlson comorbidity index group, immunosuppressed state, department of hospitalization, nosocomial infection, and appropriate empiric antibiotic treatment. The final cohort consisted of n=638 episodes due to missing data on septic shock from one study site (Seville). ST was modelled as a categorical variable with all other STs used as reference. Virulence cluster was modelled as a categorical variable with the largest cluster, Cluster E (n=192), used as reference.

Abbreviations: Observations (obs), Sequence Type (ST), Confidence Interval (CI)

References

1. Andrews, S. *et al.* FastQC. *A quality control tool for high throughput sequence data* 370, (2010).
2. Krueger, F. Trim galore. *A wrapper tool around Cutadapt and FastQC to consistently apply quality and adapter trimming to FastQ files* 516, (2015).
3. Bankevich, A. *et al.* SPAdes: a new genome assembly algorithm and its applications to single-cell sequencing. *Journal of computational biology* 19, 455–477 (2012).
4. Jolley, K. A., Bray, J. E. & Maiden, M. C. J. Open-access bacterial population genomics: BIGSdb software, the PubMLST.org website and their applications [version 1; peer review: 2 approved] . *Wellcome Open Res* 3, (2018).
5. Camacho, C. *et al.* BLAST+: architecture and applications. *BMC Bioinformatics* 10, 1–9 (2009).
6. Katoh, K. & Standley, D. M. MAFFT multiple sequence alignment software version 7: improvements in performance and usability. *Mol Biol Evol* 30, 772–780 (2013).
7. Price, M. N., Dehal, P. S. & Arkin, A. P. FastTree: computing large minimum evolution trees with profiles instead of a distance matrix. *Mol Biol Evol* 26, 1641–50 (2009).
8. Yu, G., Smith, D. K., Zhu, H., Guan, Y. & Lam, T. T. ggtree: an r package for visualization and annotation of phylogenetic trees with their covariates and other associated data. *Methods Ecol Evol* 8, 28–36 (2017).
9. Hyatt, D. *et al.* Prodigal: prokaryotic gene recognition and translation initiation site identification. *BMC Bioinformatics* 11, 1–11 (2010).
10. Buchfink, B., Reuter, K. & Drost, H.-G. Sensitive protein alignments at tree-of-life scale using DIAMOND. *Nat Methods* 18, 366–368 (2021).
11. Chen, L. *et al.* VFDB: a reference database for bacterial virulence factors. *Nucleic Acids Res* 33, D325-8 (2005).
12. Sayers, S. *et al.* Victors: a web-based knowledge base of virulence factors in human and animal pathogens. *Nucleic Acids Res* 47, D693–D700 (2019).
13. Kursa, M. B. & Rudnicki, W. R. Feature Selection with the Boruta Package. *J Stat Softw* 36, 1–13 (2010).
14. Gu, Z., Gu, L., Eils, R., Schlesner, M. & Brors, B. “Circlize” implements and enhances circular visualization in R. (2014).
