# Supplementary material for: Genomic virulence markers are associated with severe outcomes in patients with Pseudomonas aeruginosa bloodstream infection
Source: Commun Med (Lond). 2024 Dec 11;4:264. doi: 10.1038/s43856-024-00696-4 (PMC11634891; doi:10.1038/s43856-024-00696-4)
Supplement: Supplementary file 2 — Reporting Summary [file 43856_2024_696_MOESM2_ESM.pdf]

nature portfolio

Corresponding author(s): John Karlsson Valik and Pontus Nauclér

Last updated by author(s): Nov 9, 2024

Reporting Summary

Nature Portfolio wishes to improve the reproducibility of the work that we publish. This form provides structure for consistency and transparency in reporting. For further information on Nature Portfolio policies, see our Editorial Policies and the Editorial Policy Checklist.

Statistics

For all statistical analyses, confirm that the following items are present in the figure legend, table legend, main text, or Methods section.

n/a	Confirmed	
☐	☒	The exact sample size (*n*) for each experimental group/condition, given as a discrete number and unit of measurement
☐	☒	A statement on whether measurements were taken from distinct samples or whether the same sample was measured repeatedly
☐	☒	The statistical test(s) used AND whether they are one- or two-sided<br>*Only common tests should be described solely by name; describe more complex techniques in the Methods section.*
☐	☒	A description of all covariates tested
☐	☒	A description of any assumptions or corrections, such as tests of normality and adjustment for multiple comparisons
☐	☒	A full description of the statistical parameters including central tendency (e.g. means) or other basic estimates (e.g. regression coefficient) AND variation (e.g. standard deviation) or associated estimates of uncertainty (e.g. confidence intervals)
☐	☒	For null hypothesis testing, the test statistic (e.g. *F*, *t*, *r*) with confidence intervals, effect sizes, degrees of freedom and *P* value noted<br>*Give P values as exact values whenever suitable.*
☒	☐	For Bayesian analysis, information on the choice of priors and Markov chain Monte Carlo settings
☒	☐	For hierarchical and complex designs, identification of the appropriate level for tests and full reporting of outcomes
☒	☐	Estimates of effect sizes (e.g. Cohen's *d*, Pearson's *r*), indicating how they were calculated

Our web collection on statistics for biologists contains articles on many of the points above.

Software and code

Policy information about availability of computer code

Data collection	Bioinformatic databases, software and version: FastQC 0.11.9, Trim Galore 0.6.1, SPAdes 3.14.1, pubMLST March 2021, BLAST+ 2.9.0+, MAFFT 7.407, FastTree 2.1.10, Prodigal 2.6.3, Diamond 2.0.4, CARD 3.1, VFDB Downloaded March 2021, Victors Downloaded March 2021, MUMmer 4.0.0, MMSeq2 13-45111, ggtree 3.8.2, and microSALT versions 2.8.12 to 3.0.1,
Data analysis	All other data management and analysis was performed using Excel (Microsoft), R version 4.3.1, and RStudio 2023.06.2. The following packages were used: Boruta 8.0.0, Caret 6.0-94, Tidyverse 2.0.0, and Circlize 0.4.15. The source code to generate the study's main results is publicly available at https://data.mendeley.com/datasets/3h9gvbzz7x/2.

For manuscripts utilizing custom algorithms or software that are central to the research but not yet described in published literature, software must be made available to editors and reviewers. We strongly encourage code deposition in a community repository (e.g. GitHub). See the Nature Portfolio guidelines for submitting code & software for further information.

Data

Policy information about availability of data

All manuscripts must include a data availability statement. This statement should provide the following information, where applicable:

- Accession codes, unique identifiers, or web links for publicly available datasets
- A description of any restrictions on data availability
- For clinical datasets or third party data, please ensure that the statement adheres to our policy

The collected P. aeruginosa strains in this study are available from the corresponding author on reasonable request with a completed materials transfer agreement. The genomes of all sequenced bacterial strains are deposited in the Sequence Read Archive (SRA), maintained by The National Center for Biotechnology Information (NCBI). A dataset featuring annotated virulence genome data, resistance phenotype data, and the source data of the main figures is publicly available at https://data.mendeley.com/datasets/3h9gvbzz7x/271. According to current regulations, sharing restrictions apply to the dataset containing clinical parameters to safeguard the confidentiality and integrity of the study participants. Full access to patient-level data will require obtaining an ethical permit from local ethical review boards, as well as entering into a formal data-sharing agreement with the study investigators and/or affiliated institutions. For inquiries related to data access for this study, please contact the corresponding author. The study protocol is available from the corresponding author upon reasonable request.

Research involving human participants, their data, or biological material

Policy information about studies with human participants or human data. See also policy information about sex, gender (identity/presentation), and sexual orientation and race, ethnicity and racism.

Reporting on sex and gender

Data on the sex of study participants were extracted from medical records. Self-reported gender and sexual orientation were not included, as this was not central to our research question. Our study encompassed all individuals experiencing monobacterial Pseudomonas bloodstream infections, without any sex or gender-specific eligibility criteria. All multivariable analyses were adjusted for sex, making our findings applicable to both males and females. Of the 773 participants, 267 (33.4%) were female, with percentages ranging from 29.8% to 38.0% across different study sites. The study received ethical approval from the Regional Ethical Review Board in Stockholm, with a waiver for participant consent, in line with national standards for similar research. No data that could be used to individually identify patients has been shared.

Reporting on race, ethnicity, or other socially relevant groupings

No individual-level data on race, ethnicity, or other socioeconomic variables were collected for or used in this study.

Population characteristics

*Number of patients: 773
*Age, Median [IQR]: 68.0 [57.0, 78.0]
*Charlson co-morbidity score, Median [IQR]: 2.0 [1.0, 4.0]
*Department of hospitalization, n (%)
 -Surgical: 119 (15.4)
 -Medical: 479 (62.0)
 -Intensive Care Unit: 174 (22.5)
*Length of hospital stay, Median [IQR]: 20.0 [9.0, 41.0]
*Source of infection, n (%)
 -Unknown: 142 (18.4)
 -Abdominal: 77 (10.0)
 -Vascular catheter associated: 130 (16.8)
 -Pulmonary: 159 (20.6)
 -Skin/soft tissue/bone/joints: 73 (9.4)
 -Urinary: 178 (23.0)
 -Other: 14 (1.8)
*Multidrug resistant P. aeruginosa, n (%): 112 (14.5)
*Extensively drug-resistant P. aeruginosa, n (%): 50 (6.5)
*Appropriate empiric antibiotic treatment, n (%): 655 (84.7)
*Septic shock at onset, n (%): 115 (18.0)
*Mortality day 7, n (%): 120 (15.5)
*Mortality day 30, n (%): 182 (23.5)

Detailed descriptive population characteristics are presented in Table 1 of the manuscript.

Recruitment

Participants were recruited retrospectively by reviewing results from the routine clinical microbiological laboratories at each participating center. All consequtive adult patients with monobacterial P. areuginosa bloodstream infection were included. Only time periods when study centres routinely saved all their P. aeruginosa strains were included. As a positive blood culture for P. aeruginosa was a prerequisite for inclusion in the study, only patients for whom a blood culture had been collected were eligible. Consequently, our results are generalizable only to this specific population. However, patients with bloodstream infections caused by P. aeruginosa are generally in critical condition with clear signs of infection. Therefore, a blood culture is typically taken in these cases, which minimizes the likelihood of testing bias. The association between virulence markes and patient outcomes were not compared with other patient groups or pathogens, further limiting the bias.

Ethics oversight

All analyses were performed in Sweden and the study was approved by the Regional Ethical Review Board in Stockholm (approval number 2015/1184-31 and 2022-02595-02) and performed in accordance with the permission. Each participating

center were required to have local ethical review board approval or comply with other local regulatory requirements for collecting human and bacterial data retrospectively for research purposes.

Note that full information on the approval of the study protocol must also be provided in the manuscript.

Field-specific reporting

Please select the one below that is the best fit for your research. If you are not sure, read the appropriate sections before making your selection.

☒ Life sciences ☐ Behavioural & social sciences ☐ Ecological, evolutionary & environmental sciences

For a reference copy of the document with all sections, see nature.com/documents/nr-reporting-summary-flat.pdf

Life sciences study design

All studies must disclose on these points even when the disclosure is negative.

Sample size	This study is observational and the sample size was determined by the number of eligible patients presenting with P. aeruginosa bloodstream infection across the six participating centers during a 7-year period. To the best of our knowledge, this constitutes the largest cohort of its kind, making it a unique dataset for analysis. The cohort comprises approximately 800 samples with an outcome prevalence of approximately 20%. The exposure to various virulence markers ranges from 10% to 90% within this cohort. Given these parameters, the study has the power to detect odds ratios ranging from 1.5 to 2.0 with a power of 0.80 and a significance level of 0.05. These detectable odds ratios provide clinically relevant prognostic information.
Data exclusions	Two exclusion criteria were applied. First, polymicrobial bloodstream infections were excluded (if other bacteria and fungi were deemed clinically significant, as determined by investigators). This was done to enhance both internal and external validity and to assess the unconfounded effect attributable to P. aeruginosa. A single co-isolation of common contaminant species such as coagulase-negative staphylococci, Micrococcus spp., Bacillus spp., or viridans streptococci was not considered a reason for exclusion. Second, recurrent episodes in the same patient were excluded to avoid issues with correlated data, as the expected prevalence of recurrence was too low to be properly addressed in the statistical analyses. Additionally, when assessing the septic shock outcome, the 134 episodes collected in Spain (Seville) were omitted due to missing data on the outcome. For the analysis of mortality outcomes, no collected episodes were excluded.
Replication	When preforming prediciton modelling using a machine learning model, the data set was divided into a development and validation set to assure replicable results in a seperate test set.
Randomization	This cohort study is observational and did not involve any intervention, precluding the possibility of randomizing participants. In order to account for variability in the data splits during the predictive modeling process, we performed 1,000 random divisions of the dataset into development and validation sets.
Blinding	The investigators were blinded to the human data throughout the laboratority analysis, and to the bacterial virulence data throughout the collection of human data. Blinding was not possible when analysing the association between bacterial virulence gene markers and patient outcomes since a combined data set is required to perform the analyses. However, the analysis plan was independet of the exposure and outcome allocation in the cohort. Since this was an explorative study, methodological descisions were primarily taken using a data driven approach and the rationale behind these desicions is clearly explained troughout the manuscript.

Reporting for specific materials, systems and methods

We require information from authors about some types of materials, experimental systems and methods used in many studies. Here, indicate whether each material, system or method listed is relevant to your study. If you are not sure if a list item applies to your research, read the appropriate section before selecting a response.

Materials & experimental systems

n/a	Involved in the study	
☒	☐	Antibodies
☒	☐	Eukaryotic cell lines
☒	☐	Palaeontology and archaeology
☐	☒	Animals and other organisms
☒	☐	Clinical data
☒	☐	Dual use research of concern
☒	☐	Plants

Methods

n/a	Involved in the study	
☒	☐	ChIP-seq
☒	☐	Flow cytometry
☒	☐	MRI-based neuroimaging

Animals and other research organisms

Policy information about studies involving animals; ARRIVE guidelines recommended for reporting animal research, and Sex and Gender in Research

Laboratory animals	No laboratory animals were used
Wild animals	No wild animals were used
Reporting on sex	Bacteria are single-celled prokaryotic organisms with asexual reproduction and cannot be assigned a gender
Field-collected samples	Available bacterial isolates were identified retrospectively using results from the routine clinical microbiological laboratories at each participating center. Retrieved isolates were sent to Karolinska University Hospital, Stockholm, Sweden using Amies transport medium in room temperature for European samples, and glycerol transport medium in frozen temperature (-80 degree Celsius) for the Australian samples. Directly upon arrival, bacterial samples were incubated overnight on Cystine-Lactose-Electrolyte-Deficient (CLED) agar, and fresh colonies were collected and stored in -80 degree Celsius. Isolates were then retrived in separete batches for DNA extraction, whole genome sequencing (Illumina) and antimicrobial susceptibility testing.
Ethics oversight	The study was approved by the Regional Ethical Review Board in Stockholm (approval number 2015/1184-31 and 2022-02595-02) and performed in accordance with the permission.

Note that full information on the approval of the study protocol must also be provided in the manuscript.
